# Differential DNA methylation with age displays both common and dynamic features across human tissues that are influenced by CpG landscape

**DOI:** 10.1186/gb-2013-14-9-r102

**Published:** 2013-09-13

**Authors:** Kenneth Day, Lindsay L Waite, Anna Thalacker-Mercer, Andrew West, Marcas M Bamman, James D Brooks, Richard M Myers, Devin Absher

**Affiliations:** 1 HudsonAlpha Institute for Biotechnology, Huntsville, AL 35806, USA; 2 Departments Cell, Developmental, and Integrative Biology, the University of Alabama at Birmingham, Birmingham, AL 35294, USA; 3 Department of Neurology, the University of Alabama at Birmingham, Birmingham, AL 35294, USA; 4 The Center for Exercise Medicine, Birmingham, AL 35294, USA; 5 The School of Medicine, Birmingham, AL 35294, USA; 6 Stanford School of Medicine, Cancer Institute, Stanford, CA 94305, USA

**Keywords:** Aging, Methylation, Muscle, Kidney, Blood, Brain, LADs, Chromatin, H3K27Me3, Sequencing

## Abstract

**Background:**

DNA methylation is an epigenetic modification that changes with age in human tissues, although the mechanisms and specificity of this process are still poorly understood. We compared CpG methylation changes with age across 283 human blood, brain, kidney, and skeletal muscle samples using methylation arrays to identify tissue-specific age effects.

**Results:**

We found age-associated CpGs (ageCGs) that are both tissue-specific and common across tissues. Tissue-specific ageCGs are frequently located outside CpG islands with decreased methylation, and common ageCGs show the opposite trend. AgeCGs are significantly associated with poorly expressed genes, but those with decreasing methylation are linked with higher tissue-specific expression levels compared with increasing methylation. Therefore, tissue-specific gene expression may protect against common age-dependent methylation. Distinguished from other tissues, skeletal muscle ageCGs are more associated with expression, enriched near genes related to myofiber contraction, and closer to muscle-specific CTCF binding sites. Kidney-specific ageCGs are more increasingly methylated compared to other tissues as measured by affiliation with kidney-specific expressed genes. Underlying chromatin features also mark common and tissue-specific age effects reflective of poised and active chromatin states, respectively. In contrast with decreasingly methylated ageCGs, increasingly methylated ageCGs are also generally further from CTCF binding sites and enriched within lamina associated domains.

**Conclusions:**

Our data identified common and tissue-specific DNA methylation changes with age that are reflective of CpG landscape and suggests both common and unique alterations within human tissues. Our findings also indicate that a simple epigenetic drift model is insufficient to explain all age-related changes in DNA methylation.

## Background

Methylation is a major biochemical alteration that governs multi-tiered epigenetic regulation of gene expression. Methylation of specific lysine resides within core histones such as H3 and H4 induce conformational modifications in chromatin structure that are associated with regulation of transcriptional activity [1]. Direct methylation of DNA itself is one foundational epigenetic modification that typically involves conversion of cytosine to 5′-methylcytosine catalyzed by DNA methyltransferases [2]. DNA methylation in adult tissues usually occurs within the context of CpG dinucleotide sequences (CpGs) clustered in regions known as CpG islands (CGIs) that are most often found proximal to promoters of housekeeping genes [3]. For the majority of genes, hypermethylation of CpG islands is linked with transcriptional silencing. During embryogenesis, DNA is passively demethylated during early cell divisions until *de novo* DNA methylation establishes the CpG methylation marks within dividing cells that guide restriction of gene expression patterns associated with tissue-specific lineages [4,5]. During maintenance of tissues, CpG methylation marks must also be maintained by DNA methyltransferases during DNA replication in dividing adult stem cells to preserve the identity and function of differentiating cell types and for self-renewal of adult stem cell populations [6-8].

The context of DNA methylation in relation to CGIs has emerged as a defining feature within genome-wide DNA methylation studies. Approximately 65 to 70% of promoters are associated with CGIs, and these promoter types are generally hypomethylated, while promoters that contain a low CpG density are hypermethylated [9,10]. Comparison of differential DNA methylation patterns between induced pluripotent stem cells and their parental fibroblasts showed an overlap of CpGs with tissue- and cancer-specific methylation patterns in regions located within 2 kb of CGIs known as CpG shores (CGSs) [11]. Intriguingly, the same methylation changes in CpGs during cellular differentiation overlap with those most frequently altered in cancer cells, and suggests that aberrant DNA methylation could be an underlying factor in the genesis of cancer stem cells [12-14]. Outside of CGIs and CGSs, including gene bodies, DNA methylation is often a characteristic of active transcription with sharp transitions in methylation between exon and intron boundaries [15,16].

Early epigenetic studies showed an effect of aging on the stability of X-linked chromosome gene inactivation [17]. An increase in DNA methylation differences between young and old monozygotic twin pairs established a strong link between phenotypic discordance, epigenetics, and aging [18]. This relationship between DNA methylation and age raises questions of how epigenetic changes may specifically influence different tissue types over time, especially in adult tissues composed mainly of postmitotic cells such as neurons and multinucleated myofibers. It has been proposed that epigenetic changes with age, including DNA methylation, may be a stochastic process of random epigenetic 'drift' [19,20]. Comparisons of DNA methylation between normal versus cancer tissue or between epithelial to mesenchymal cell transitions during development suggest shared methylation 'noise' within the same CpGs is indicative of some level of modulated cellular plasticity [21]. Furthermore, subtle methylation changes may be functionally important, as has been shown in the adult brain where stimulus-induced methylation may be related to neuronal plasticity [22]. All of these findings suggest a highly dynamic epigenome, even within fully differentiated, post-mitotic cells.

Here we compare DNA methylation alterations with age across brain, blood, kidney, and skeletal muscle tissues with array-based DNA methylation profiling that interrogated 26,486 autosomal CpGs covering approximately 14,000 genes. We found a significant number of age-associated CpGs (ageCGs) near genes that are commonly involved in developmental processes, transcription, and morphogenesis. The majority of these CpGs are increasingly methylated with age (positive ageCGs) and positioned within CGIs. However, ageCGs that were found within non-CGIs mostly displayed decreasing methylation with age (negative ageCGs) and were enriched in tissue-specific ageCGs. Skeletal muscle exhibited negative ageCGs that were found within non-CGIs and associated with genes that code for components required for myofiber contraction. In three of the tissues, ageCGs were enriched in genes that are not highly expressed in that tissue, according to Illumina Body Map 2.0 data. However, the strongest relationship between ageCGs and genes that are expressed was within skeletal muscle. Genes near negative ageCGs generally had higher expression levels than positive ageCGs. Analysis of tissue-specific chromatin states generated using Roadmap Epigenomics project data showed marks associated with bivalent and active chromatin encompassing positive or negative ageCGs, respectively. Negative ageCGs are also generally closer in genomic distance to tissue-specific CTCF binding sites. We also find an enrichment of ageCGs within lamina-associated domains (LADs) that are involved in attachment of chromatin to the inner nuclear membrane. We further validated the observed array-based age effects in the kidney by targeted capture bisulfite sequencing of select promoter regions and demonstrate a more widespread effect across multiple CpGs in these age-sensitive regions. Our data show that aging has both dynamic and common influences on DNA methylation.

## Results

### Common and distinct features of age-dependent methylation among tissues

We bisulfite converted genomic DNA isolated from human blood, brain, kidney, and skeletal muscle tissue samples collected from different ages (Additional file 1). To identify ageCGs across tissues, we performed genome-wide DNA methylation profiling using Illumina beadchips that query 26,486 autosomal CpGs. Beta scores (percentage methylation; β-scores) were calculated and quality filtered followed by ComBat batch normalization across samples for each tissue separately (Materials and methods) [23]. We used a linear regression model in each tissue separately to test for associations between β-scores and age, with gender as a covariate. The resulting *P-*values were adjusted by the false discovery rate method (*q*, Benjamini and Hochberg method). CpGs that exhibited *q* < 0.05 were considered ageCGs (Additional files 2, 3, 4, 5, 6). CpGs that did not exhibit an age effect (*q* > 0.5) were defined as nonageCGs used for further comparisons to ageCGs for each tissue.

**Figure 1:**
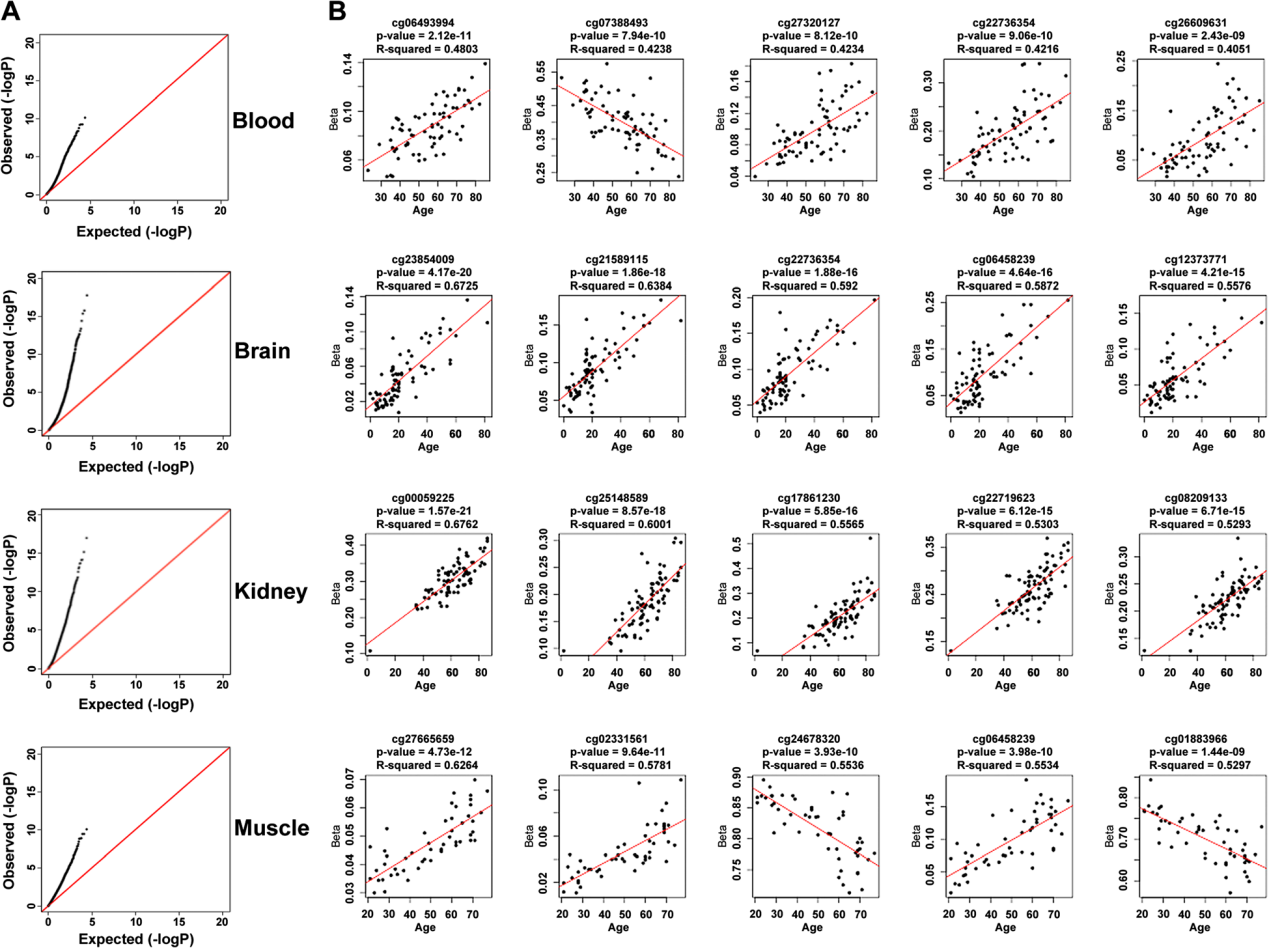
**Linear regression results showed an association between DNA methylation and age across four human tissues. (A)** Uniform quantile-quantile (Q-Q) plots of -log *P-*values from linear regression tests for associations between β-score and age within blood, brain, kidney, and skeletal muscle tissue samples. **(B)** Scatterplots for the top five strongest associations between β-score and age across each of the four tissues. Illumina CpG ID, *P-*value, and R-squared results are depicted above each scatterplot.

Uniform quantile-quantile (Q-Q) plots of negative log *P-*values from our linear regression results revealed many *P-*values that were smaller than expected and implied strong, widespread associations between age and DNA methylation in each tissue (Figure 1A). The majority of ageCGs showed a positive correlation (increasing DNA methylation with age; positive slope) as shown by representative regression plots of the five most significant ageCGs in each tissue (Figure 1B). Some of our samples were from both normal and disease subjects, but none of these disease phenotypes were strongly correlated with DNA methylation patterns in our samples when covariates for disease phenotypes were used to correct for any differences between groups (Additional file 7: Figure S1). Neighboring ageCGs that were proximal to the same gene region within a tissue showed approximately 91% agreement in their slope direction with age irrespective of their genomic distance in base pairs from one another (Additional files 8, 9, 10, 11). However, the absolute value of the differences (delta) between the slopes of the regression models at neighboring ageCGs was generally much smaller when they were within a distance of 100 bp from each other compared to those that were at least 500 bp apart (Additional file 7: Figure S2).

We compared our list of ageCGs from blood and brain with independent studies of age-dependent methylation. Approximately 48% of age-related, differentially methylated regions in whole blood that were validated with independent samples using sorted CD4^+^ and CD14^+^ cells were also found in our ageCG list from blood [24]. Seven out of seven Methylation27 CpGs from a recent report that used blood-specific, age-dependent methylation to build a predictive model of human aging rates from Methylation450 array data (89 CpGs total in the model; 7 CpGs are also found on the Methylation27 array) were in our ageCG list from blood and exhibited the same slope trend with age [25]. In another recent study that reported the top 100 CpGs that showed changes in methylation in dorsolateral prefrontal cortex brain regions with age across individuals greater than 10 years old using Methylation27 arrays, 68 of these CpGs were also found in our brain ageCG list [26]. We found that the slopes determined for these CpGs between our study and Numata *et al*. [26] were highly correlated (Pearson correlation, r = 0.92; Additional file 7: Figure S3).

**Figure 2:**
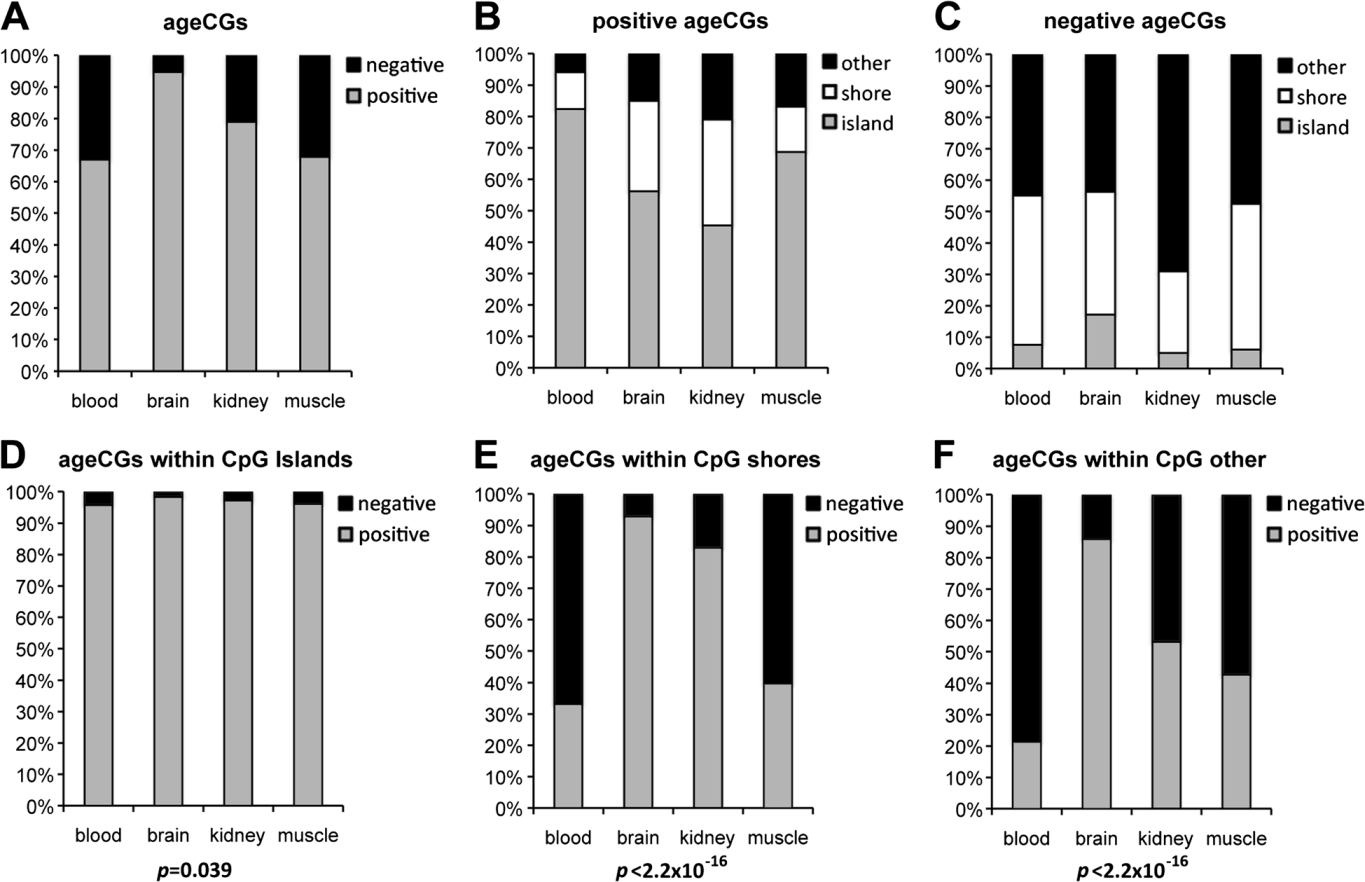
**Bar graphs displaying the percentages of ageCGs classified by CpG context and positive or negative association with age across four human tissues. (A)** Percentage of total ageCGs that exhibited a positive or negative association with age in each tissue. **(B**,**C)** Percentage of positively associated **(B)** and negatively associated ageCGs **(C)** positioned within CpG islands, shores, or other. **(D**-**F)** Percentages of positively or negatively associated ageCGs each classified within CpG islands **(D)**, CpG shores **(E)**, or CpG other contexts **(F)**. *P-*values are below bar graphs that resulted from Pearson’s Chi-squared tests between number of positive or negative associations across tissues in each respective CpG context.

**Figure 3:**
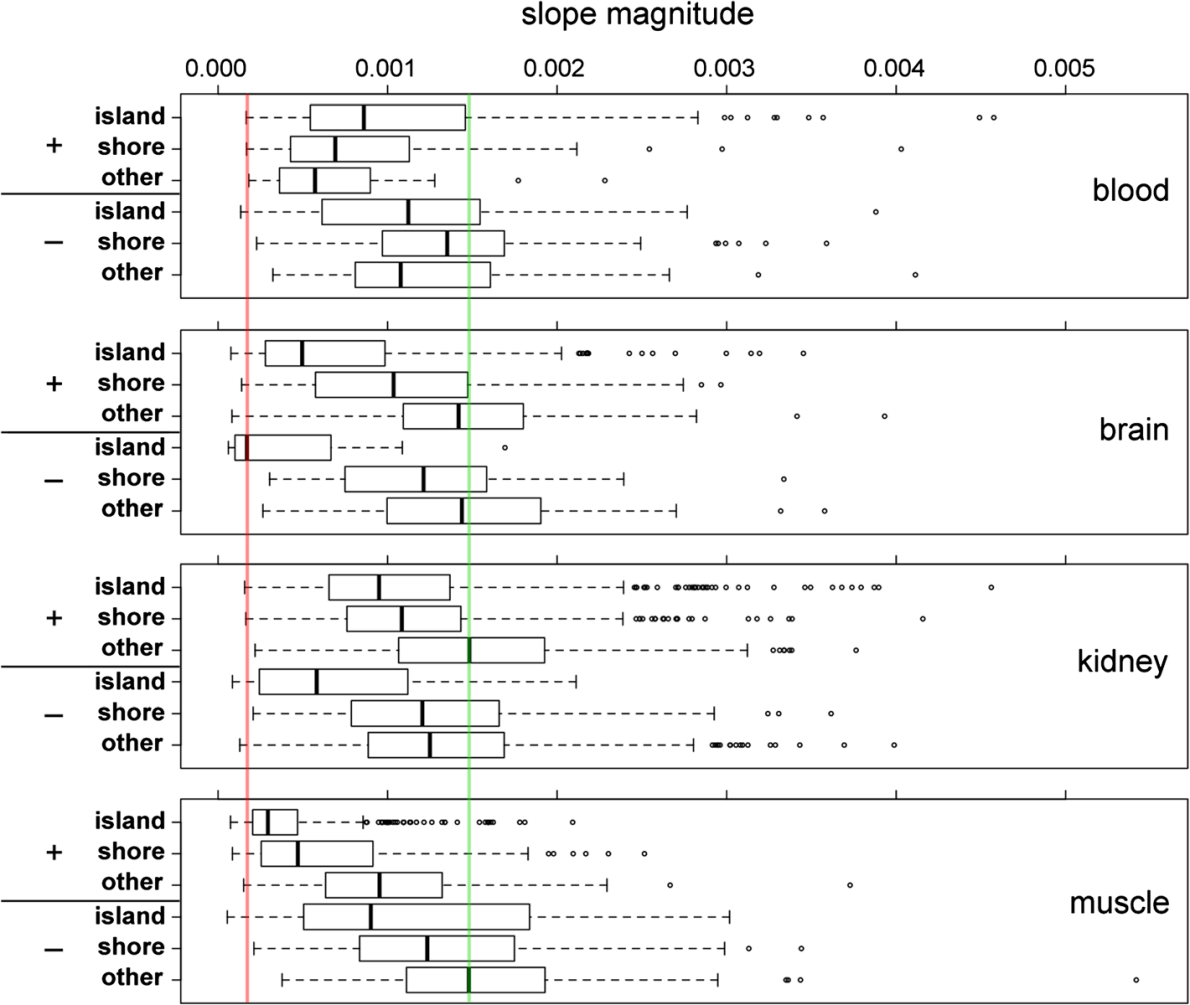
**Boxplots of slope magnitude grouped according to positive (+) or negative (-) ageCGs and CpG context across four tissues.** Vertical red and green lines mark the smallest and largest median slope magnitude, respectively, across all four tissues. A significant three-way interaction between CpG context, tissue type, and positive or negative slope trend was found among slope magnitude values (ANOVA on Box-Cox power transformed slope values, *P* = 5.4 × 10^-5^).

We classified ageCGs according to positive or negative correlation with age (positive or negative ageCGs), and whether they were positioned within a CGI or non-CGI context. Pearson’s Chi-squared tests confirmed an association between CGI or non-CGI context and positive or negative ageCGs, respectively, in all tissues (*P* < 2.2 × 10^-16^; Figure 2A-C). Profiling of ageCGs located within CGIs, CGSs, or CpG other (CpGs located outside of islands or shores, CGOs) according to UCSC genome browser definition of CGIs showed that ageCGs from each tissue generated CpG context profiles that were different from non-ageCGs [3] (Table 1). A greater percentage of ageCGs was found within CGIs compared to non-ageCGs with the exception of kidney ageCGs, which were enriched within CGOs. The strongest differences in the proportions of positive or negative ageCGs between tissues were found in CGSs and CGOs (Pearson’s Chi-square tests, *P* < 2.2 × 10^-16^; Figure 2D-F). The majority of ageCGs within CGIs were hypomethylated across all samples and all tissues (β-scores < 0.3; Additional file 7: Figure S4). Comparisons of the 10 youngest and oldest samples confirmed that the greatest tissue-specific variation in methylation involved negative ageCGs outside of CGIs (Additional file 7: Figure S5). Negative ageCGs were also generally larger in magnitude than positive ageCGs (Additional file 7: Figure S6). We normalized slope magnitude distributions using Box-Cox power transformations to compare slope magnitudes across tissues and found a significant three-way interaction between CpG context, tissue type, and slope trend (ANOVA, *P* < 5.4 × 10^-5^; Figure 3). These results suggest that common age-dependent methylation (mostly increasing) occurs primarily within CGIs, and tissue-specific patterns (mostly decreasing) reside outside of CGIs, with different rates of methylation both within and among tissues.

**Table 1:** **CpG context profiles of ageCGs and non-ageCGs across tissues**^**a**^

	**AgeCGs**^**b**^	**Non-ageCGs**^**c**^	***P*-value**^**d**^
**Blood:**			
**Island**	533 (57.7%)	8,106 (45.2%)	
**Shore**	217 (23.5%)	5,915 (33.0%)	2.48 × 10^-13^
**Other**	174 (18.8%)	3,923 (21.9%)	
**Brain:**			
**Island**	646 (54.1%)	7,486 (49.4%)	
**Shore**	351 (29.4%)	5,132 (33.9%)	3.16 × 10^-3^
**Other**	198 (16.6%)	2,532 (16.7%)	
**Kidney:**			
**Island**	987 (36.8%)^e^	6,005 (46.2%)	
**Shore**	859 (32.1%)	4,086 (31.5%)	<2.2 × 10^-16^
**Other**	833 (31.1%)	2,898 (22.3%)	
**Muscle:**			
**Island**	476 (48.6%)	5,978 (41.6%)	
**Shore**	242 (24.7%)	5,038 (35.1%)	3.14 × 10^-10^
**Other**	262 (26.7%)	3,353 (23.3%)	

^a^Percentage of total CpGs for each tissue indicated in parentheses.

^b^Age-associated CpGs (*q* < 0.05 with linear regression model).

^c^Non-age-associated CpGs (*q* > 0.5 with linear regression model).

^d^Resulting *P*-values from Pearson’s Chi-squared tests (3x3 contingency tables containing ageCGs and non-ageCGs).

^e^AgeCGs from kidney tissue are less frequently found in islands compared to other tissues.

To further validate our identification of common and tissue specific methylation changes with age, we compared the intersection of the same ageCGs that overlapped between at least two tissues. As expected, most overlapping ageCGs between tissues were found in CGIs with increasing methylation (Additional files 7: Figure S7). There was an overlap of 29 CpGs among all four tissues, but at least half of all ageCGs in each tissue were not common with other tissues (Additional file 7: Figure S8). We found a maximum overlap of only four CpGs among all four tissues by chance by comparing overlap of the same number of randomly selected CpGs from the Methylation27 array as the number of ageCGs found for each tissue and verified that the overlap is an effect of aging (*P* < 1 × 10^-4^). Overlapping ageCGs in the 10 youngest samples showed the least variation in methylation between tissues for those with increasing methylation in CGIs, as expected (all β-scores < 0.1; Additional file 7: Figure S9). Analysis of significant Gene Ontology (GO) term enrichments (*q* < 0.05) for positive and negative ageCGs linked with genes (ageCGs/genes) for each tissue showed that approximately 77% of GO terms for positive ageCGs/genes overlapped between at least 2 tissues, but only approximately 10% of GO terms for negative ageCGs/genes overlapped (only between kidney and blood). Shared terms for positive ageCGs/genes were related to morphogenesis and transcription, and unique terms for positive ageCGs/genes in a single tissue were often closely related to terms that overlapped (Table 2; Additional files 12, 13, 14, 15, 16, 17, 18). Regardless of increasing or decreasing methylation, half of all shared GO terms between two tissues (two-way shared) were between kidney and blood. The only shared terms for negative ageCGs/genes were between kidney and blood in terms related to immune response. Skeletal muscle negative ageCGs/genes showed the strongest tissue-specific enrichments in GO terms related to muscle contraction (Table 2).

**Table 2:** **Summary of Gene Ontology terms associated with positive and negative ageCGs affiliated with genes across tissues**^**a**^

**GO term**^**b**^	**Tissue**	**Count**	***P*-value**	***q*-value**	**Fold**
**Positive unique**					
GO:0030054 - cell junction	Blood	42	5.2 × 10^-10^	7.0 × 10^-7^	3.1
GO:0048754 - branching morphogenesis of a tube	Brain	18	1.6 × 10^-7^	3.0 × 10^-4^	5.0
GO:0048878 - chemical homeostasis	Kidney	87	4.9 × 10^-15^	9.0 × 10^-12^	2.6
GO:0003713 - transcription coactivator activity	Muscle	15	6.6 × 10^-7^	1.0 × 10^-3^	5.6
**Positive two-way shared**					
GO:0000902 - cell morphogenesis	Blood	37	2.3 × 10^-14^	4.0 × 10^-11^	4.9
GO:0003002 - regionalization	Brain	41	5.3 × 10^-15^	9.6 × 10^-12^	4.6
GO:0000902 - cell morphogenesis	Kidney	73	2.9 × 10^-16^	6.1 × 10^-13^	3.1
GO:0003712 - transcription cofactor activity	Muscle	23	3.2 × 10^-9^	4.9 × 10^-6^	4.9
**Positive three-way shared**					
GO:0007267 - cell-cell signaling	Blood	56	8.9 × 10^-28^	1.6 × 10^-24^	6.5
GO:0007267 - cell-cell signaling	Brain	64	4.3 × 10^-21^	7.8 × 10^-18^	4.2
GO:0030182 - neuron differentiation	Kidney	117	4.5 × 10^-37^	8.2 × 10^-34^	4.1
GO:0016563 - transcription activator activity	Muscle	29	5.0 × 10^-10^	7.5 × 10^-7^	4.2
**Positive four-way shared**					
GO:0005886 - plasma membrane	Blood	169	1.2 × 10^-23^	1.6 × 10^-20^	2.1
GO:0031226 - intrinsic to plasma membrane	Brain	119	1.7 × 10^-36^	2.5 × 10^-33^	3.8
GO:0031226 - intrinsic to plasma membrane	Kidney	209	1.3 × 10^-60^	1.9 × 10^-57^	3.7
GO:0006350 - transcription	Muscle	102	1.5 × 10^-18^	2.6 × 10^-15^	2.6
**Negative unique**^**c**^					
GO:0006952 - defense response	Kidney	58	5.8 × 10^-30^	1.0 × 10^-26^	6.8
GO:0005887 - integral to plasma membrane	Kidney	68	2.0 × 10^-22^	2.7 × 10^-19^	4.0
GO:0006954 - inflammatory response	Kidney	30	7.3 × 10^-15^	1.3 × 10^-11^	6.5
GO:0009611 - response to wounding	Kidney	39	2.2 × 10^-14^	3.8 × 10^-11^	4.6
GO:0005886 - plasma membrane	Kidney	138	9.1 × 10^-14^	1.2 × 10^-10^	1.8
GO:0006936 - muscle contraction	Muscle	15	1.5 × 10^-11^	2.5 × 10^-8^	13.0
GO:0003012 - muscle system process	Muscle	15	7.6 × 10^-11^	1.3 × 10^-7^	11.5
GO:0006941 - striated muscle contraction	Muscle	10	1.3 × 10^-9^	2.2x10^-6^	21.3
GO:0015629 - actin cytoskeleton	Muscle	15	3.6 × 10^-6^	4.7 × 10^-3^	4.8
GO:0003009 - skeletal muscle contraction	Muscle	5	5.0 × 10^-6^	8.3 × 10^-3^	44.3
**Negative two-way shared (kidney and blood only)**^**d**^					
GO:0006955 - immune response	Kidney	66	2.6 × 10^-32^	4.5 × 10^-29^	6.3
GO:0031226 - intrinsic to plasma membrane	Kidney	69	2.1 × 10^-22^	2.7 × 10^-19^	4.0
GO:0005576 - extracellular region	Kidney	105	1.8 × 10^-15^	2.3 × 10^-12^	2.2
GO:0044459 - plasma membrane part	Kidney	95	7.5 × 10^-13^	9.9 × 10^-10^	2.1
GO:0002684 - positive regulation of immune					
system process	Kidney	24	1.3 × 10^-10^	2.3 × 10^-7^	5.5

^a^DAVID v6.7 was used with all genes represented on the Methylation27 beadchip as background and gene lists associated for each tissue according to minimum age-associated *P*-value and positive or negative ageCGs. Enrichment tests were done independently for each tissue. Complete results are shown in supplemental data. All results shown are below *q* < 0.05 as generated by DAVID analysis [27,28].

^b^Results were first sorted by shared GO terms among tissues according to whether they overlapped among all four tissues (four-way), among three tissues (three-way), between two tissues (two-way), or unique in only one tissue. GO terms were then sorted by tissue and lowest *q-*values. The top GO term is shown for each tissue using this sorting scheme.

^c^The top five genes affiliated with unique negative ageCGs in each tissue shown here do not include genes from brain because results did not meet *q* < 0.05for blood tissue see below.

^d^All genes affiliated with negative ageCGs in blood were shared with kidney. Results are only shown here for kidney tissue.

### CpGs affiliated with tissue-specific gene expression are protected from common methylation changes with age

Given our observation of tissue-specific age effects, we hypothesized that tissue-specific gene expression might influence the likelihood of age-dependent DNA methylation. We used Illumina Human Body Map 2.0 Project RNA-Seq data and determined fragments per kilobase of exon per million fragments mapped (FPKM) for Methylation27 array genes across white blood cells, kidney, brain, and skeletal muscle tissues. We compared the enrichment of ageCGs/genes and non-ageCGs/genes according to their proximity to non-expressed (FPKM < 0.05) or expressed (FPKM > 0.25) genes for each tissue. With the exception of skeletal muscle, ageCGs/genes were enriched in non-expressed genes compared to non-ageCGs/genes (Table 3). Blood held the fewest ageCGs/genes affiliated with gene expression, and ageCGs positioned within CGIs and CGSs were affiliated with non-expressed genes with the exception of skeletal muscle, which showed no significant difference in expression between ageCGs and non-ageCGs positioned within CGSs (Table 3). Kidney and skeletal muscle ageCGs/genes within CGOs were significantly associated with tissue-specific gene expression compared to non-ageCGs/genes. These results suggest that the further ageCGs were positioned from CGIs, the more likely these ageCGs were positioned near tissue-specific, expressed genes.

**Table 3:** **AgeCGs and nonageCGs affiliated with gene expression within tissues classified by CpG context**^**a**^

	**AgeCGs/genes**^b^		**Non-ageCGs/genes**^c^		***P*-value**^d^
	**Expressed**	**Non-expressed**	**Expressed**	**Non-expressed**	
**Blood**					
All	271 (46.2)	315 (53.8)	4610 (78.4)	1270 (21.6)	<2.2 × 10^-16^ *
Island only	121 (36.1)	214 (63.9)	2251 (89.0)	277 (11.0)	<2.2 × 10^-16^ *
Shore only	101 (72.7)	38 (27.3)	1610 (83.5)	317 (16.5)	1.5 × 10^-3^ *
Other only	49 (43.8)	63 (56.2)	749 (52.6)	676 (47.4)	0.09
**Brain**					
All	591 (78.8)	159 (21.2)	4404 (89.3)	528 (10.7)	3.5 × 10^-16^ *
Island only	328 (79.4)	85 (20.6)	2243 (97.0)	70 (3.0)	<2.2 × 10^-16^ *
Shore only	184 (85.6)	31 (14.4)	1564 (95.0)	82 (5.0)	1.2 × 10^-7^ *
Other only	79 (64.8)	43 (35.2)	597 (61.4)	376 (38.6)	0.53
**Kidney**					
All	1209 (77.4)	354 (22.6)	4046 (89.9)	456 (10.1)	<2.2 × 10^-16^ *
Island only	402 (71.2)	163 (28.8)	2089 (97.5)	54 (2.5)	<2.2 × 10^-16^ *
Shore only	440 (85.8)	73 (14.2)	1362 (95.0)	71 (5.0)	1.1 × 10^-11^ *
Other only^e^	367 (75.7)	118 (24.3)	595 (64.3)	331(35.7)	1.6 × 10^-5^ *
**Muscle**					
All^f^	515 (77.2)	152 (22.8)	2784 (71.8)	1095 (28.2)	4.2 × 10^-3^
Island only	258 (78.4)	71 (21.6)	1262 (87.3)	184 (12.7)	5.2 × 10^-5^ *
Shore only	142 (86.1)	23 (13.9)	1065 (82.0)	233 (18.0)	0.24
Other only	115 (66.5)	58 (33.5)	457 (40.3)	678 (59.7)	1.6 × 10^-10^ *

^a^Expressed genes, FPKM > 0.25; non-expressed genes, FPKM < 0.05. Parentheses indicate percentage of total in each CpG context category.

^b,c^Genes in proximity to an ageCG or non-ageCG according to the smallest age-associated *P*-value for representative genes in each tissue.

^d^Resulting *P*-values from Pearson’s Chi-squared tests between numbers of ageCGs and non-ageCGs within each classification.

^e^AgeCGs/genes found within CGOs from kidney showed a significantly greater number of expressed genes compared to non-ageCGs in kidney.

^f^Muscle was the only tissue that showed a greater number of total ageCGs/genes expressed compared to non-ageCGs/genes regardless of CpG contextthis same effect was observed within the CGO category.

*Significant *P*-values meet Bonferroni correction for multiple testing.

Other nonparametric tests of nonzero expression levels between ageCGs/genes and nonageCGs/genes produced similar results to our expressed/non-expressed thresholds (Additional file 7: Figure S10). With the majority of ageCGs exhibiting increased methylation within CGIs (associated with non-expression), we also compared expression levels connected with positive and negative ageCGs/genes. Negative ageCGs/genes showed higher gene expression levels compared to positive ageCGs/genes, with kidney showing the weakest difference (Additional file 7: Figure S10). Based on our findings of strong overlap between GO terms generated for kidney and blood, especially immune-related terms for negative ageCGs/genes, we analyzed significant differentially expressed genes (using Student’s *t*-tests built into Cufflinks software) between blood and kidney for kidney positive and negative ageCGs/genes [29]. Negative kidney ageCGs/genes were enriched in blood-specific gene expression levels, and the opposite effect was observed for blood versus muscle, and blood versus brain (Additional file 7: Figure S11). These data suggested that more kidney-specific methylation (as assessed by kidney-specific ageCGs/genes) might be found within positive ageCGs compared to other tissues. Therefore, we used a more stringent approach to identify the strongest tissue-specific ageCGs/genes by isolating ageCGs/genes that uniquely held strong age associations in one tissue (*q* < 0.05) while these same CpGs were considered not significant (non-ageCGs; *q* > 0.5) in the other three tissues (unique ageCGs/genes). We used the smallest age-associated *P-*value for representative genes affiliated with CpGs in each tissue to determine unique ageCGs/genes. There were 28, 32, 144, and 132 unique ageCGs/genes in blood, brain, kidney and muscle tissues, respectively (Additional file 19). Among all 4 tissues, 41 ageCGs/genes showed a shared, strong age effect (*q* < 0.05 for ageCGs within the same gene identified in all 4 tissues).

Unique ageCGs/genes were significantly enriched in expression compared to shared ageCGs (with the exception of brain), and the strongest enrichment was observed in skeletal muscle (Table 4). As anticipated, approximately 90% or greater of shared ageCGs/genes compared to approximately 30% of unique ageCGs/genes were positive ageCGs within CGIs. No clear trend was associated with ageCGs/gene expression and CpG context (Additional file 7: Figure S12). Intercepts from regression results (that is, predicted methylation level at age 0) among all tissues across shared ageCGs/genes were very similar regardless of affiliated gene expression (hypomethylated <0.2), but blood and kidney both had significantly greater slope magnitudes for these shared ageCGs/genes compared to brain and muscle (ANOVA, *P* < 0.003; Additional file 7: Figure S13). These results suggested some variability in rates of methylation between tissues within shared ageCGs. There was greater variability in intercept values and slope magnitudes among tissues within unique ageCGs (Additional file 7: Figure S13). Unique ageCGs/genes affiliated with expressed genes exhibited a trend towards hypomethylation (median intercepts < 0.2), and with the exception of blood, a range of intercepts that extended toward hypermethylation were affiliated with non-expressed genes. We found the unique ageCGs/genes *PLAT*, *PROM1*, *KCNJ1*, and *PCK1* to be uniquely and significantly expressed only in kidney, and the *MB* gene in skeletal muscle (Additional files 20, 21, 22, 23). Altogether, different approaches to isolate common and tissue-specific changes with age revealed the importance of local gene expression on the propensity of a CpG to show a common age-effect among tissues.

**Table 4:** **Number of unique and shared ageCGs/genes affiliated with genes expressed within tissues**^**a**^

	**Unique ageCGs/genes**^b^		**Shared ageCGs/genes**^c^		***P*-value**^d^
	**Expressed**	**Non-expressed**	**Expressed**	**Non-expressed**	
**Blood**	13 (65.0)	7 (35.0)	8 (22.9)	27 (77.1)	0.003 *
**Brain**	16 (72.7)	6 (27.3)	27 (75.0)	9 (25.0)	0.766
**Kidney**	82 (78.8)	22 (21.2)	17 (56.7)	13 (43.3)	0.019
**Muscle**	84 (88.4)	11 (11.6)	11 (42.3)	15 (57.7)	3.40 × 10^-6^ *

^a^Expressed genes, FPKM > 0.25; non-expressed genes, FPKM < 0.05. Parentheses indicate percentage of total in each CpG context category.

^b^Unique ageCGs/genes had *q* < 0.05 from linear regression results in one tissue and *q* > 0.5 for the same affiliated gene in the other three tissues.

^c^Shared ageCGs/genes had *q* < 0.05 in all four tissues.

^d^Resulting *P*-values from Fisher’s exact tests between the number of unique and shared, expressed or non-expressed ageCGs/genes within tissues.

*Significant *P*-values meet Bonferroni correction for multiple testing.

### Regional chromatin landscapes affiliated with common and tissue-specific methylation changes with age

**Figure 4:**
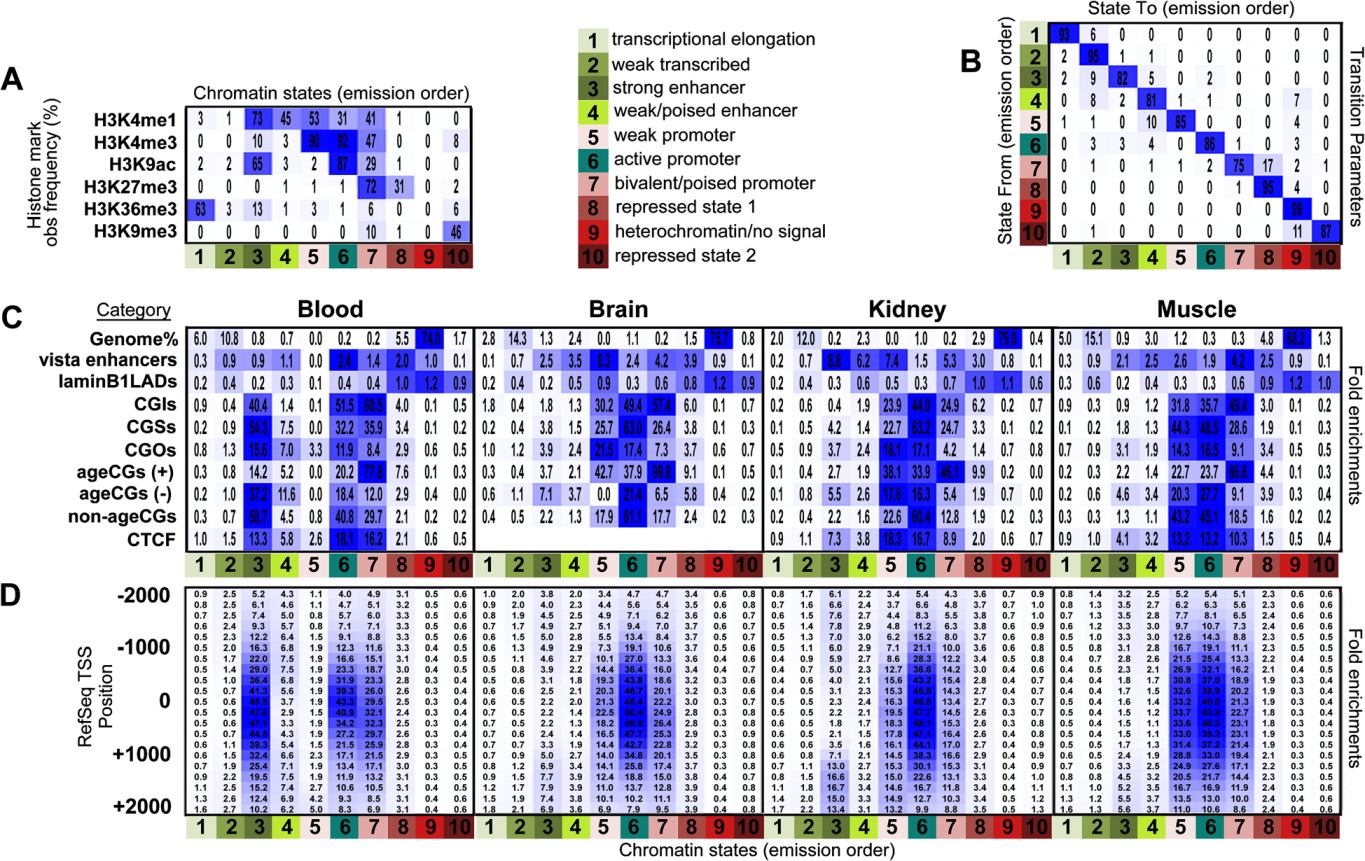
**Jointly learned tissue-specific chromatin states across four human tissues and functional enrichments within positive and negative ageCG positions.** Ten input chromatin states using Roadmap Epigenome ChIP-seq data for six histone modifications across four human tissues were used with ChromHMM software. **(A)** Heatmap/table shows learned emission parameters based on genome-wide combinations of histone marks. Values indicate observed frequencies of histone modifications found at genomic positions corresponding with chromatin states. **(B)** Transitional parameter heatmap/table shows probabilities of transitions between states (multiplied by 100). Rows show the 'from' chromatin state and columns show the 'to' chromatin state, that is, a 17% probability that chromatin state 7 transitioned into state 8. **(C)** Heatmap/table depicts percentage of the genome for each chromatin state (topmost row) and relative fold functional enrichments of genome category (that is, vista enhancers, lamin B1 laminB1lads, CpGs within CGIs, CGSs, CGOs, positive (+) and negative (-) ageCGs and non-ageCGs). Enrichments for chromatin states underlying ChIP-seq CTCF binding sites were determined using ENCODE data for CD14^+^ and CD20^+^ cells (merged peaks blood), kidney tissue, and myotubes (brain data not available). Overlap enrichments were determined separately for each tissue using tissue-specific segmentation files generated from the jointly learned model. Values across rows indicate relative fold enrichment, and blue color scale is based on subtraction of the minimum value in the row divided by the maximum row value for each tissue separately (vertical black lines divide table enrichments per tissue). **(D)** Neighborhood enrichments for RefSeq transcriptional start site annotations (TSS) within chromatin states determined using default 0-based anchor coordinates for each start site position. Fold enrichment values and color scale are according to rows. In all panels, the lower axis shows chromatin state colored to match chromatin state descriptions (1 to 10).

We were interested in chromatin signatures affiliated with positive and negative ageCGs across all four tissues. We automated development of learned chromatin state parameters and chromatin state assignments with publicly available ChIP-seq data sets for histone modifications from the Roadmap Epigenomics Project (peripheral blood mononuclear cells, brain hippocampus, fetal kidney, and adult skeletal muscle) using ChromHMM software [30]. The repressive H3K27Me3 mark played a critical role in discerning diversity in states; without this mark included in the model only 2 to 3 main states were observed even when using between 5 and 10 input states. With model parameters that assigned 10 chromatin states across tissues, we determined relative fold enrichments for positive and negative ageCGs (Figure 4A-C). Comparison of enrichments of positive and negative ageCGs within chromatin states across tissues in general showed tissue-specific differences in underlying chromatin states connected with age-dependent methylation that were most noticeable in blood. Positive ageCGs were most enriched in the bivalent/poised promoter defined state containing repressive H3K27Me3 marks present with other active H3K4Me1, H3K4Me3, and H3K9Ac marks (Figure 4A, C) [31-33]. The bivalent promoter state and repressed state 1 frequently transitioned between each other, with the repressed state containing only the H3K27Me3 mark by itself (Figure 4B). Both active and bivalent promoter states were strongly enriched in RefSeq transcriptional start site positions (Figure 4D).

Negative ageCGs were enriched in weak/active promoter and enhancer-related chromatin states compared to positive ageCGs. Enhancer-related states 3 and 4 had the highest level of H3K4Me1 mark with the lowest co-occurrence of H3K4Me3, which is linked to active or poised enhancers [34,35]. The underlying defined chromatin states for positive and negative ageCGs were consistent with gene expression results even when we stratified by expression (data not shown). We also found that CTCF ChIP-seq peaks from ENCODE data (CD14^+^/CD20^+^ cells, kidney tissue, myotubes) were enriched in similar chromatin states as negative ageCGs (Figure 4C, brain unavailable). We found very few ageCGs that overlapped with CTCF binding sites, and CTCF sites were generally greater than 5 kb in genomic distance from ageCGs compared to non-ageCGs, again with the exception of skeletal muscle, which showed the opposite effect (Pearson’s Chi-squared tests blood, *P* = 0.024, kidney, *P* = 5.88 × 10^-12^, muscle, *P* = 0.002). With the exception of kidney, negative ageCGs were closer to CTCF binding sites than positive ageCGs (Additional file 7: Figure S14). Positive ageCGs alone were significantly further away from a CTCF site compared to non-ageCGs, with the exception of skeletal muscle (>5 kb Pearson’s Chi-squared tests blood, *P* = 0.001, kidney, *P* = 2 × 10^-7^, muscle, not significant). Only muscle negative ageCGs exhibited significantly closer proximity to CTCF sites compared to non-ageCGs (*P* = 2.5 × 10^-5^). We found a negative correlation between the distance of a CTCF binding site to a Methylation27 CpG and FPKM values (data not shown). As expected, the relationship of the CTCF binding site distance was similar to our gene expression results for positive and negative ageCGs.

We also identified an enrichment of ageCGs within LADs [36]. Approximately 75% of these ageCGs were positive ageCGs, and 20 to 30% of total ageCGs were within LADs, except for muscle (Additional file 7: Figure S15). LADs-associated ageCGs were found within chromatin states enriched in H3K27Me3 compared to nonageCGs. Previous studies demonstrated that H3K27Me3 enriched chromatin is usually located closer to LAD edges [36]. We oriented ageCGs within LADs according to gene direction to a LAD edge (going toward or away from a LAD). Distributions of the genomic distance between a CpG to a LAD edge (normalized by the LAD length) were bifurcated, and suggested a nearness of LAD-bound CpGs to LAD edges. CTCF binding sites are also associated with LAD edges. Tissue-specific, CTCF binding sites also oriented according to gene direction showed close distance to a LAD edge (also normalized by LAD length) for sites both upstream and downstream of CpGs. The distributions of ageCG distance to a LAD edge did not vary much from nonageCGs, although slightly more nonageCGs were centrally located. Therefore, a fraction of increasing, age-dependent methylation is associated with LAD boundaries.

### Methylation changes with age extend beyond single CpGs

**Figure 5:**
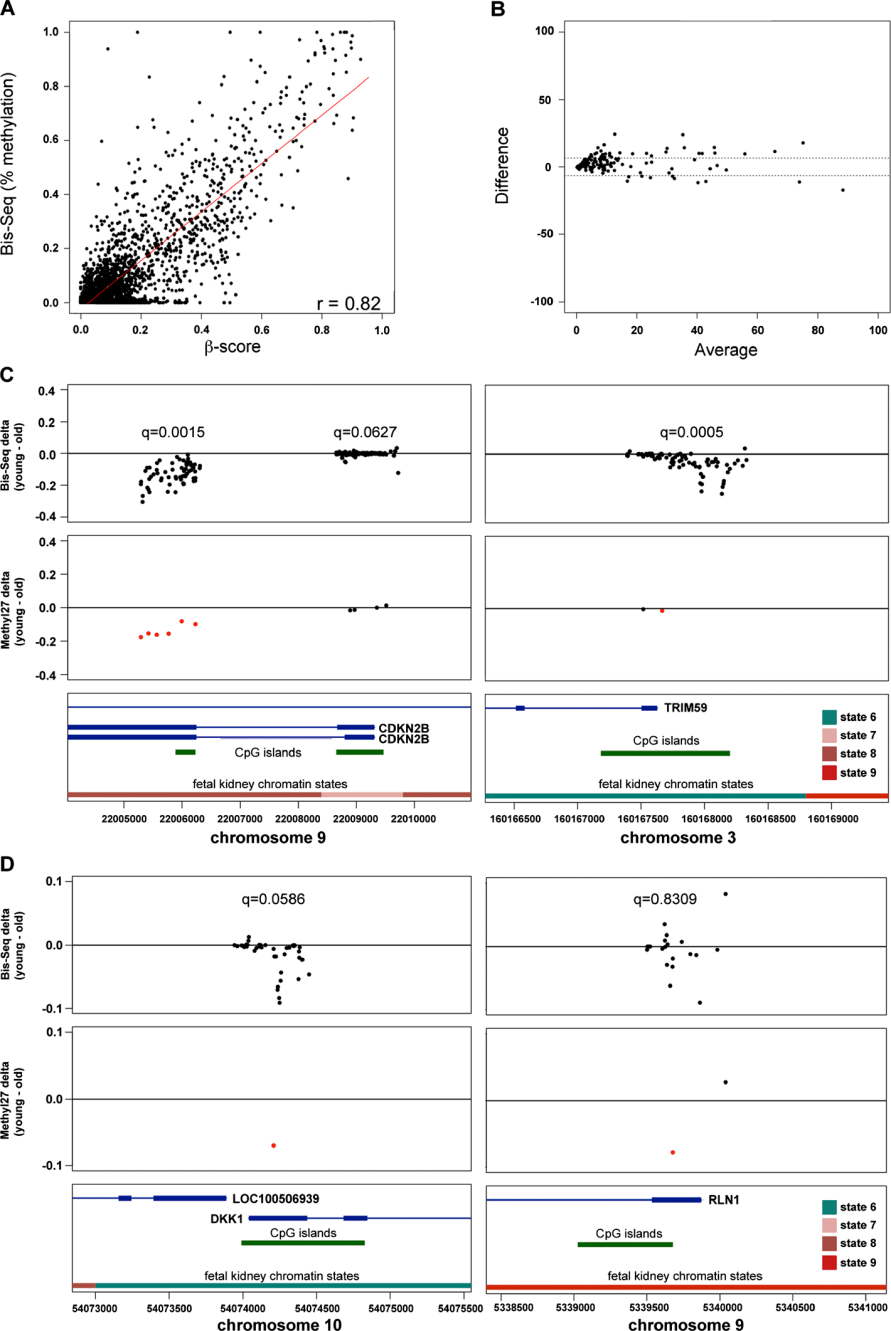
**Validation of ageCGs by targeted capture and bisulfite sequencing of genomic regions encompassing ageCG sites. (A)** Scatterplots of percentage methylation by bisulfite sequencing (Bis-seq) versus Methylation27 β-score and correlation of methylation values between the two methods across 19 kidney samples used for validation. **(B)** A representative Bland-Altman plot for comparison of Bis-seq and Methylation27 methylation values. Points depict the average percentage methylation between both methods plotted against the differences in methylation between the methods. Dotted lines show limits of agreement (average difference ± 1.96 standard deviation of the difference). **(C**, **D)** Comparison of methylation delta values (median percentage methylation of young minus old samples) at CpGs covered by Bis-seq (top panels) and Methylation27 arrays (middle panels). Red points represent ageCGs, and delta values shown for Bis-seq are only for the 9 youngest and 10 oldest samples. False discovery rate (q) values indicate significance level of a widespread age effect by linear mixed model results with Bis-seq data at these target regions. The DKK1 target just missed the significance threshold (q < 0.05), and the RLN1 target demonstrates an example region that contained abrupt changes in direction of delta values among neighboring CpGs. Bottom panels depict labeled gene regions (blue boxes, exons blue lines, introns), CpG islands (green boxes), and fetal kidney chromatin states (colors correspond with definitions in Figure 4). Greater details and examples of all Bis-seq targets are available in Additional files 7 and 24.

We were also interested in how extensive methylation changes were around identified age-sensitive sites by Methylation27 arrays. We developed custom biotinylated RNA capture probes and bisulfite sequenced (Bis-seq) approximately 83 sites representing 77 gene regions/promoters and validated ageCGs in the 9 youngest and 10 oldest kidney samples (Additional file 24; Materials and methods). Out of 3,734 sequenced CpGs, 128 covered between both methods were well correlated with regard to percentage methylation across all samples, with individual Pearson correlation values that ranged from r = 0.93 to 0.71 (Figure 5A). Approximately 86% of CpGs across all samples differed by less than 15% methylation, and 71% differed by less than 10% methylation between method platforms. As expected, agreement within 95% confidence limits, as shown by Bland-Altman plot (depicts the differences in agreement between two methods), was relatively stronger for hypomethylated CpGs (<20% methylated) compared to hypermethylated CpGs (Figure 5B). Calculation of delta between median percentage methylation values at each CpG for young and old groups validated both ageCGs and non-ageCGs within target sites observed by arrays along with neighboring CpGs not covered by arrays (Figure 5C, D). Most sites showed increasing methylation with age, but some sites showed fluctuations between positive and negative deltas that were also observed by Methylation27 arrays. Often we observed that the magnitude of delta values at neighboring CpGs were greater than the ageCGs originally identified by the Methylation27 array.

Linear mixed models were used to test the association of widespread methylation changes with age at single targets across multiple CpGs. An autoregressive correlation structure was used in which CpGs located in close proximity to one another were considered more strongly correlated than CpGs that were further apart. Out of 60 separate targets that contained a strong age effect (q < 0.05) for single CpGs determined by linear regression model by array data across all kidney samples, 38 showed a strong widespread age effect across multiple CpGs by Bis-seq (Figure 5C; Additional file 7: Figure S16, Additional file 24). Some targets barely missed our significance threshold (q < 0.05) such as *DKK1* presumably because the age effect was isolated at a few CpGs within the target, and other targets showed oscillation in the direction of the age effect between closely neighboring CpGs such as *RLN1* (Figure 5D; Additional file 7: Figure S17 and Figure S18, Additional file 24). We included 14 negative control targets where we did not expect to find an age effect by Bis-seq, but 3 of these targets did show a widespread age effect (Additional file 7: Figure S19 and Figure S20, Additional file 24). There were also nine Bis-seq targets we intentionally captured where there were no Methylation27 CpGs or where Methylation27 CpGs were removed by quality filtering, and four of these targets showed an age association by Bis-seq (Additional file 7: Figure S21, Additional file 24). Lastly, we also found cases where we captured regions where an age effect bordered groups of CpGs that showed no difference in methylation between young and old (Additional file 7: Figure S22).

We found our linear mixed models were limited for detection of widespread age effects within the regions where the direction of the effect oscillated abruptly between neighboring CpGs as described above. In order to investigate these more complicated regions, additional mixed models were constructed to test for an interaction between age and chromosomal position, which would indicate a change in the slope of the age effect at different regions within the target. These models, however, did not lead to the identification of any widespread age-associated targets beyond what was already identified in the primary mixed model. Overall, Bis-seq revealed a more widespread age effect across the majority of promoter region targets with coverage of neighboring CpGs near our identified ageCGs, and confirmed our array findings.

## Discussion

Our parallel analysis of age-dependent methylation in four human tissues demonstrates specific epigenetic patterns near ageCG sites identified across adult tissues. Throughout our analysis, we found that skeletal muscle was unique as this tissue showed age-dependent methylation linked with tissue-specific expressed genes and proximity to CTCF binding sites. Our results further indicate distinct landscapes of negative ageCGs that are enriched in tissue-specific methylation differences compared to positive ageCGs, which were more often shared among tissue types (Table 5). In an analysis of 1,413 CpGs among tissues that did not include skeletal muscle, a study similar to ours also found a relationship between increasing and decreasing methylation with age and CpG context [37]. Our analysis of DNA methylation in brain is consistent with other studies that showed enrichment of larger slope magnitude and negative correlation with age in non-CGIs, but the majority of the age-effect was increasing methylation with a relatively smaller slope magnitude typically within CGIs [26,38]. Other studies also found age-associated methylation changes using Methylation27 arrays within normal tissues such as dermal fibroblasts [39], whole blood [13,24], CD4^+^ T cells and CD14^+^ monocytes [13], hematopoietic progenitor cells [7], and mesenchymal stromal and stem cells [40,41]. Common and tissue-specific, age-related changes were also identified in various rodent tissues [42,43]. Recent whole genome bisulfite sequencing comparisons of cord blood from newborns and CD4^+^ T cells from centenarians also demonstrated hypomethylation in non-CGIs near tissue-specific genes and hypermethylation within CGIs [44]. A predictive model for age based on Methylation450 data from blood samples showed that this model was less predictive for other tissues, and tissue-specific predictive models each contained different CpG markers [25]. Therefore, the mechanistic basis for both common and tissue-specific DNA methylation changes over time is unclear.

**Table 5:** Descriptive features and enrichments of differentially methylated sites with age across human blood, brain, kidney, and skeletal muscle tissues

**Positive ageCGs**	**Negative ageCGs**
Within CGIs	Within CGSs, CGOs
Generally hypomethylated	Generally hypermethylated
Smaller relative slope magnitude	Larger relative slope magnitude
Greater enrichment in H3K27Me3	Less enrichment in H3K27Me3
More shared sites across tissues	More unique sites across tissues
Genes with lower relative FPKM	Genes with higher relative FPKM
Near development-related genes	Near tissue-specific regulated genes
Generally further from CTCF sites	Generally closer to CTCF sites
Enriched within LADs	Not enriched in LADs

Increased DNA methylation near non-expressed genes related to developmental processes, and decreased methylation within tissue-specific, differentiation-related genes that are expressed may suggest an age effect that is driven by the activity of progenitor populations. The traditional role of maintenance DNA methylation activity occurs within the context of cell division as DNA methyltransferase 1 (DNMT1) detects hemimethylated DNA [45]. DNA methylation errors with age were shown to be associated with increased methylation within mitotic cells, while non-mitotic tissue remained unchanged [46]. In a highly mitotic tissue such as epidermis, DNMT1 was expressed in epidermal progenitors, lost during differentiation, and required for sustained repression of differentiation [47]. DNMT1 also was required to repress *CDKN2A* and *CDKN2B* genes, two cyclin-dependent kinase inhibitor genes that may inhibit adult stem cell self-renewal [47,48]. *CDKN2A*/*B* are two major sites of age-dependent methylation that we observed in multiple tissues. DNMT1 depletion or Gadd45-dependent, DNA demethylation led to upregulation of differentiation-related genes such as actin, tropomyosin, and myosin heavy chain genes in skeletal muscle [47]. Therefore, it is possible that age-related methylation changes we observe could be signatures of adult stem cell activity. In mice, maintenance of DNA methylation was required for hematopoietic stem cell self-renewal [49]. Analysis of DNA methylation within hematopoietic stem cells showed age-dependent demethylation within a subset of CpGs near myeloid-specific, differentiation-related genes [7]. Transplanted hematopoietic stem cells from old mice also demonstrated a bias toward the myeloid lineage [50]. In summary, maintenance of tissues by adult stem cells may be one explanation of age-dependent methylation, and will require future emphasis of investigation within sorted cell populations.

Some methylation changes with age may also be signatures of minor changes in tissue composition. Inflammation and fibrotic infiltration of tissues are common features in solid tissues such as kidney and muscle with age [51-54]. Interestingly, negative kidney ageCGs produced many GO terms affiliated with immune response and more blood-specific gene expression compared to positive ageCGs/genes linked with relatively more kidney-specific gene expression. These results may be reflective of immune cell infiltration in kidney. Muscle-specific GO terms linked to negative ageCGs could, therefore, be associated with skeletal muscle fiber type changes. Previous analyses of gene expression in muscle and kidney tissues along with histology of skeletal muscle demonstrated tissue-remodeling changes with age [54,55]. We have also found histological changes in skeletal muscle fibers with age in conjunction with gene expression studies from the same muscle samples used to isolate DNA for this study (manuscript in preparation). The use of nuclei enrichment methods to isolate native differentiated cell types from infiltrating cells and adult stem cells may help to more clearly define the possibility of genuine age-related methylation changes in non-mitotic cells.

Age-related DNA methylation may also be explained by potential changes in chromatin structure. In addition to our study, other groups have identified enrichment of age-dependent methylation within repressive or bivalent chromatin [24,41]. A recent study showed DNMT1 occupancy within hypermethylated gene bodies of transcribed genes and bivalent chromatin states [56]. It has been proposed that breakdown of chromatin boundaries at the transcriptional start and end sites may lead to aberrant spreading of methylation into promoters and reduction of gene body methylation [56]. Therefore, it is conceivable that partitioning of positive ageCGs in bivalent or repressed chromatin, and negative ageCGs enriched in tissue-specific methylation changes in enhancer-related chromatin could be related to chromatin boundary breakdown and aberrant DNMT1 activity. CTCF is associated with boundary formation between active and repressed chromatin [57]. Variable CTCF occupancy was also shown to be influenced by differential DNA methylation [58]. We found very few CTCF binding sites directly covering ageCGs, but sites were generally closer to negative ageCGs near genes that were expressed, and proximity of CTCF to a Methylation27 CpG was associated with higher FPKM. Therefore, it is possible that tissue-specific, decreasing methylation changes with age could be linked to nearby CTCF binding. The enrichment of some ageCGs within LAD edges may also suggest age-related epigenetic influences on chromatin boundary structure in relation to the nuclear membrane. Some studies have shown that nuclear architecture could be altered during normal aging beyond just modifications connected with premature aging syndromes [59]. The methyl CpG binding protein MeCP2 was shown to interact with the lamin B receptor and may connect DNA methylation with nuclear architecture [60]. Similar to our findings, whole-genome bisulfite sequencing showed that approximately 36% of identified age-associated, differentially methylated regions were within LADs [44]. Altogether, future studies of DNMT1 binding and the influence of chromatin boundaries may provide explanations for some aspects of age-dependent methylation.

While our results indicate that age-dependent methylation changes cannot be completely explained by stochastic alterations, we have not eliminated the potential role of stochastic mechanisms at work. The majority of ageCGs (except skeletal muscle) were mostly associated with genes that are not expressed within these tissues, and may suggest that these ageCG sites are subject to epigenetic drift more often within silenced or bivalent chromatin. It has been proposed that the chromatin domains surrounding these age-dependent methylated sites may contribute to epigenetic 'control' of cellular plasticity as a result of noise that could connect epigenetic changes with age to pre-neoplastic conditions [13,21]. We found that the majority of shared methylation changes across tissues were increasing with age in CGIs. Whole genome bisulfite sequencing of newborns and centenarians also showed that decreasing methylation with age outside of CGIs (also enriched near tissue-specific genes) is more prevalent than increasing methylation [44]. Thus, our results yielding greater numbers of positive ageCGs that were often shared among tissues may be due to emphasis of Methylation27 CpG selection within CGIs. Greater coverage of CpGs across the genome by bisulfite sequencing therefore should expose more widespread tissue-specific regions associated with aging.

## Conclusions

Our results indicate that age-dependent methylation changes cannot be completely explained by stochastic events, although positive ageCGs are enriched for common effects among tissues that could reflect stochastic processes. In contrast, sites containing negative ageCGs are enriched in tissue-specific differences in methylation, so we suspect that some aspect of age-dependent methylation is regulated. Our findings concerning DNA methylation and age in skeletal muscle and kidney are of major interest. Skeletal muscle showed the least overlap in total ageCGs with other tissues, had the strongest association of ageCGs related to tissue-specific gene expression, and generally showed a difference throughout our comparisons to blood, brain, and kidney in our analysis. Kidney tissue showed the largest number of ageCGs outside of CGIs and many negative ageCGs were shared with blood-specific genes related to immune response. Therefore, we conclude that there are multifaceted influences of aging on whole tissue DNA methylation changes.

## Materials and methods

### Ethics statement regarding human tissue samples

All tissue samples were collected according to protocols and guidelines at their respective institutions. Iron deficient anemia buffy coat samples were commercially available and collected by Conversant Bio (Huntsville, AL, USA). Blood DNAs were isolated using the Agencourt Genfind v2 kit (Beckman Coulter, Indianapolis, IN, USA) at HudsonAlpha Institute for Biotechnology (HAIB). Frozen post-mortem brain tissues from Brodmann area 19 of the cerebral cortex were obtained from the Harvard Brain Bank, the NICHD Brain Bank at the University of Maryland, and the Autism Tissue Program. All brain bank specimens were designated as not human subjects (NHS) by relevant institutional review boards of both sending and receiving institutes. Resected normal kidney tissue samples and DNAs were collected at Stanford in accordance with approved institutional review board protocol (6208, Panel: 8) for the purpose of a larger paired sample study with kidney tumors (manuscript in preparation). Signed patient consent for use of kidney tissue states that clinical and pathological data (including patient age) can be associated with their clinical samples and tissue would otherwise be discarded after processing for clinical care. Consent forms are stored at Stanford University and available for review according to local, state, and federal regulations. Vastus lateralis muscle samples were collected at the Department of Physiology and Biophysics and the Center for Aging at the University of Alabama at Birmingham as part of an approved institutional review board exempt, de-identified tissue bank. Muscle DNA was isolated at HAIB and the University of Alabama at Birmingham using a Fastprep automated homogenizer (MP Biologicals Solon, OH, USA) and DNeasy tissue kit (Qiagen, Germantown, MD, USA) optimized for DNA extraction from skeletal muscle.

### HumanMethylation27 BeadChip data analysis

Isolated genomic DNA (0.5 to 1 μg) was bisulfite converted according to manufacturer’s protocol using the EZ-96 DNA Methylation Kit (Zymo Research Corporation, Irvine, CA, USA). Bisulfite-converted genomic DNA was whole-genome amplified and hybridized to Infinium HumanMethylation27 BeadChips according to standard protocol (Illumina, San Diego, CA, USA). Green and red signal intensity data for autosomal CpGs, negative control probe data, and detection *P-*values were exported from Genome studio (Illumina) and imported into R for data handling and analysis. Signal intensity data were divided between probes that used either green or red detection (each unmethylated/unconverted and methylated bead type is designed to query a single CpG using single color detection). Median green and red background fluorescence intensities calculated from negative control probes were subtracted from probe signal intensity data. Signal intensities that did not have detection *P-*values (<0.01) that were significant above the average background for negative control probes in addition to samples and probe sets that had greater than 10% missing values were removed from the data set. Percentage methylation values (β-scores) were calculated by dividing probe B intensity (detection of unconverted, methylated DNA) by probe B plus probe A (detection of converted, unmethylated DNA) intensities. Negative β-scores (values where the methylated probe was originally below median background intensity) were adjusted to a value of zero and β-scores >1 (values where the unmethylated probe was originally below median background intensity) were adjusted to a value of 1. Calculations of β-scores by Illumina-based software, Genome Studio, included the addition of a correction factor of 100 in the denominator to prevent values that are below 0 or above 1. However, we found that the addition of this correction factor distorted some β-scores for probes that yielded lower intensities so we opted to exclude 100 in the β-score calculation (Additional file 7: Figure S23). Raw microarray data have been submitted and are available from the National Center for Biotechnology Information (NCBI) Gene Expression Omnibus (GEO) repository (accession number [GEO: GSE49909]).

β-Scores were also filtered based on target probe sequences that did not uniquely map to a single site in the human genome, or that contained a SNP with a minor allele frequency of 3% or greater (according to HapMap data) within 15 bp of the queried CpG. To adjust β-scores for batch effects among beadchips for each tissue type separately, missing values were imputed and β-scores were normalized by nonparametric empirical Bayes framework method within an R package called ComBat [23,61], without the inclusion of any additional covariates. Imputed values were used during normalization only because ComBat does not permit missing values in the data set, and imputed values were set back to missing after normalization. ComBat normalization was run separately for each tissue because tissue sample collections and arrays were run at different times as separate experiments. Therefore, batch effect across arrays would be completely confounded with tissue, and methylation differences between tissues would be suppressed in the normalization process. Accordingly, we felt it was inappropriate to normalize tissues together. We found that background intensity subtraction followed by normalization across beadchips by ComBat method produced the highest correlation among β-scores for 23 technical replicates from brain tissue for probes containing a low to high range of CpG content (Additional file 7: Figure S24). To determine age associations, linear regression analysis was performed using separate models for each tissue with β-scores as outcomes. Resulting *P-*values were adjusted by the false discovery rate method. CpGs that exhibited a *q*-value <0.05 were considered to be ageCGs. Comparisons between tissues were made in parallel with regression model results rather than β-scores generated from each tissue because data from each tissue were normalized and analyzed separately.

### Targeted bisulfite sequencing

Forward and reverse primer sets were designed to flank ageCGs and nonageCGs as determined by beadchip results (Additional file 24). All forward primers contained integrated T7 promoter sequences. Probe templates were amplified by PCR using 25 ng of blood genomic DNA (Conversant) with AmpliTaq polymerase (Applied Biosystems, Foster City, CA, USA) using the following reaction conditions: 95°C for 2 minutes, 30 to 35 cycles of 95°C for 30 s, 55°C for 30 s, 72°C for 2 minutes, and 72°C for 5 minutes. Reactions that had a low yield were reassembled using Titanium Taq polymerase (Clontech, Mountain View, CA, USA with the following conditions: 95°C for 1 minute, 30 to 35 cycles of 95°C for 30 s, 68°C for 2 minutes, and 68°C for 3 minutes. PCR products were purified using Qiaquick PCR spin columns (Qiagen) and re-amplified using the same PCR conditions with Titanium Taq. Final PCR products were vacuum purified using 96-well PCR purification plates (Qiagen) and quantified by the PicoGreen method (Invitrogen, Carlsbad, CA, USA). PCR products were diluted and pooled equally (approximately 125 ng each) in groups of eight to nine, and biotinylated capture probes were synthesized by *in vitro* transcription using a T7 MAXIscript kit (Ambion, Woodlands, TX, USA) with Biotin-11-UTP (Life Technologies, Grand Island, NY, USA). Unincorporated nucleotides were removed using NucAway gel filtration columns (Ambion). Individual probe pools were quantified by Qubit RNA fluorometry (Invitrogen), and mixed equally in a final probe pool based on RNA concentration.

Multiplexed Illumina sequencing libraries containing inline methylated barcoded adapters (iXpressGenes, Huntsville, AL, USA) were constructed from 1 to 1.5 μg kidney DNA samples that represented the 9 youngest (35 to 47 years old) and 10 oldest (74 to 86 years old) individuals that were previously analyzed on HumanMethylation27 BeadChips. Genomic DNAs were sheared to a size range of 100 to 500 bp with a Bioruptor XL sonicator (Diagenode, Denville, NJ, USA) with a refrigerated recirculator containing 50% ethylene glycol solution. Samples were sheared using four cycles of 30 s on and 30 s off for 10 minutes each. Genomic DNAs were end-repaired, adenylated, and ligated according to the standard protocol. Barcoded libraries were quantified by Qubit HS DNA fluorometry (Invitrogen) and pooled in 4-plex (125 ng each).

In preparation for solution hybridizations, stock human Cot-1 DNA (Invitrogen) was ethanol precipitated, resuspended in nuclease-free water and 20 μg was mixed with library DNAs. Library DNA solutions were concentrated to 7.5 μl by SpeedVac (Thermo Scientific, Asheville, NC, USA). Probe solution was prepared by dilution of 14 ng of biotinylated probe in a final volume of 6 μl containing nuclease-free water with 20 U of Superasin (Ambion). Library DNA solutions were heated on a thermal cycler to 95°C for 5 minutes followed by 65°C for 5 minutes and mixed with 13 μl of 2× hybridization buffer (10× SSPE, 10× Denhardts, 10 mM EDTA, and 0.2% SDS) and 6 μl of probe solution that were pre-warmed to 65°C for 5 minutes. After 24 h incubation at 65°C, 20 μl of pre-washed MyOne Steptavidin C1 Dynabeads (Invitrogen) were mixed with hybridizations. After frequent agitation of beads with hybridizations for 30 minutes, beads were collected on a magnet and washed twice in 0.5 ml wash buffer 1 (1× SSC, 0.1% SDS) at room temperature for 15 minutes each with frequent mixing. Beads were collected and washed three times in 0.5 ml pre-warmed wash buffer 2 (0.1× SSC, 0.1% SDS) at 65°C for 10 minutes each on a heat block. Beads were resuspended in 40 μl EB buffer and captured libraries were bisulfite converted according to manufacturer’s protocol suggested for small amounts of fragmented DNA using the Epitect Bisulfite Kit (Qiagen). DNA was eluted in 40 μl of EB buffer and a second round of bisulfite conversion was performed. Final bisulfite converted, captured 4-plex libraries were amplified by PCR in a 50 μl reaction volume containing 5 μl 10× PCR buffer, 2 μl of 50 mM MgCl_2_, 2.5 μl of 10 mM dNTPs, 1 μl of 25 μm PE1 and PE2 amplification primer mix, 5 μl of 5 M betaine, and 1 μl of Platinum Taq polymerase (Invitrogen) using the following reaction conditions: 98°C for 1 minute, 22 cycles of 95°C for 30 s, 62°C for 3 minutes. PCR reactions were cleaned up and 4-plex library concentrations were determined by Qubit HS DNA fluorometry (Invitrogen). Final 12-plex libraries were assembled by pooling equal concentrations of three 4-plex libraries. Final Illumina library preparation and sequencing was performed according to standard protocol for 2 × 72 bp paired-end runs on an Illumina GAIIx sequencer.

The pass filter sequence attributed to each barcode was demuxed and assigned to each individual using the Barcodes software [62], which was also used to design inline barcoded adapter sequences. Human hg19 DNA sequence was bisulfite converted *in silico* and a reference for mapping was built in Bowtie [63]. In preparation for mapping (solution hybridization captured library forward strand), forward reads were bisulfite converted *in silico*, and reverse complement sequence from reverse reads was made before conversion. Bowtie was used for mapping converted reads to the converted reference with -ff and --norc options and SAM file output parameters. Following alignment, mapped genomic coordinates associated with SAM files were reestablished with the original unconverted sequence reads. Duplicate reads were removed, sorted by coordinate with respect to reference, and BAM files were created for GATK input [64]. Pileups were assembled across unique reads and percentage methylation was calculated at individual CpGs. Base reads exhibiting phred quality scores below 30 were filtered from the analysis and only final percentage methylation values with 20× minimum depth coverage were used for correlations with β-scores generated from beadchip analysis. Median percentage methylation for all 10 old samples were subtracted from the median percentage methylation of the 9 young samples at individual CpGs to generate delta values for genome plots depicting bisulfite sequenced targets.

Linear mixed models were used to model association of methylation changes with age at multiple CpGs within 83 bisulfite-sequenced targets using a single model for each target. An autoregressive correlation structure was used, providing for a model in which CpGs located in close proximity to one another were more strongly correlated than CpGs that were further apart. Models were run using the R package 'nlme' with the correlation structure 'corCAR1', which represents an AR(1) autoregressive correlation structure for continuous covariates. Fixed effects included in the model were age and chromosomal mapping position. Mixed models were fit on data from 84 targets. For one gene (ELOVL4), two targets overlapped in covered CpGs, so the data from both targets were combined into a single model, leading to 83 models rather than 84.

### Gene expression and chromatin state analysis

Human tissue RNA-Seq data were generated at Illumina and are publicly available through the Human Body Map 2.0 Project [65]. These libraries were constructed from poly-A selected mRNAs isolated from kidney, brain, white blood cells, and skeletal muscle. One run for each tissue of 2 × 50 bp paired-end data sets were used for gene expression analysis. Sequence reads were mapped with TopHat [66], and expression FPKM values and differences among tissues across genes were determined by Cufflinks [29] with hg19 human gtf reference. Publicly available ChIP-seq bed files for all histone modifications, including ChIP-seq input control, were downloaded for peripheral blood mononuclear primary cells, brain middle hippocampus, fetal kidney day122, and skeletal muscle at the Roadmap Epigenomics project website at the NCBI [67]. ChromHMM is a publicly available java-based software package that binarizes histone modification signatures as either present or absent and integrates multiple chromatin datasets across tissues to develop learned chromatin states via a multivariate hidden Markov model [68]. An input of 10 chromatin states was used with default parameters. Overlap enrichment of chromatin states with genomic coordinates of interest included our hg19 genomic coordinates of ageCGs. CTCF ChIP-seq peak data sets for CD20^+^ cells, monocytes (Monocytes-CD14 + _RO01746), kidney tissue (kidney_OC) and myotubes (HSMMtube) were downloaded from the ENCODE ChIP-seq experiment matrix [69]. Genomic distance from CpGs or LAD edges to the middle of CTCF peak coordinates were calculated in R. LAD genomic coordinates were downloaded from Guelen *et al*. [36].

## Abbreviations

ageCG: Age-associated CpG; Bis-seq: Bisulfite sequenced; bp: Base pair; CGI: CpG island; CGO: CpG other; CGS: CpG shore; FPKM: Fragments per kilobase of exon per million fragments mapped; GO: Gene ontology; HAIB: HudsonAlpha institute for biotechnology; LAD: Lamina-associated domain.

## Competing interests

The authors declare that they have no competing or conflicting interests.

## Authors’ contributions

KD isolated DNA from blood and muscle tissue, analyzed methylation array data, performed all experiments for array validation and analyzed data, drafted the manuscript, and conceived the study. LLW analyzed methylation array data with emphasis in statistical testing, bioinformatics, programming, and helped to draft the manuscript. ATM and MB collected vastus lateralis muscle samples, ATM helped with DNA isolation from muscle tissue, study design, and analysis. AW collected brain tissue samples, isolated DNA, and participated in study design. JB collected kidney tissue samples and isolated DNA. RMM contributed to study design. DA analyzed methylation array data and validation data, designed primers for validation studies, helped to draft the manuscript, and conceived the study. All authors read and approved the final manuscript.

## Supplementary Material

Additional file 1 Description of samples used in this study. Click here for file

Additional file 2 Linear regression model results for ageCGs identified in each tissue separately. Click here for file

Additional file 3 UCSC Genome browser track for blood ageCGs. Click here for file

Additional file 4 UCSC Genome browser track for brain ageCGs. Click here for file

Additional file 5 UCSC Genome browser track for kidney ageCGs. Click here for file

Additional file 6 **UCSC Genome browser track for muscle ageCGs.** Click here for file

Additional file 7 Supplemental Figures S1 to S24. Click here for file

Additional file 8 Multiple ageCGs affiliated with the same genes in blood. Click here for file

Additional file 9 Multiple ageCGs affiliated with the same genes in brain. Click here for file

Additional file 10 Multiple ageCGs affiliated with the same genes in kidney. Click here for file

Additional file 11 Multiple ageCGs affiliated with the same genes in muscle. Click here for file

Additional file 12 Top 5 unique and shared GO terms across tissues. Click here for file

Additional file 13 Unique GO terms across tissues for positive ageCGs/genes. Click here for file

Additional file 14 Unique GO terms across tissues for negative ageCGs/genes. Click here for file

Additional file 15 Shared GO terms between at least two tissues for positive ageCGs/genes. Click here for file

Additional file 16 Shared GO terms between at least three tissues for positive ageCGs/genes. Click here for file

Additional file 17 Shared GO terms between all four tissues for positive ageCGs/genes. Click here for file

Additional file 18 Shared GO terms between at least two tissues for negative ageCGs/genes. Click here for file

Additional file 19 Unique and shared ageCGs/genes across all tissues. Click here for file

Additional file 20 Blood unique ageCGs/genes only expressed within blood. Click here for file

Additional file 21 Brain unique ageCGs/genes only expressed within brain. Click here for file

Additional file 22 Kidney unique ageCGs/genes only expressed within kidney. Click here for file

Additional file 23 Muscle unique ageCGs/genes only expressed within muscle. Click here for file

Additional file 24 **Target regions and primer sets used to synthesize probes for capture bisulfite sequencing to validate ageCGs identified by Methylation27 arrays, and linear mixed model results for each target containing coefficients, *P*-values, and *q*-values.** Also included are Methylation27 CpGs that were covered by Bis-seq, associated IlluminaIDs, and *q*-values resulting from linear regression with age across all CpGs, and indication as to whether these same CpGs were significantly associated with age with linear regression results only across the same 19 samples that were bisulfite sequenced. Click here for file
