# Supplementary material for: Differential DNA methylation with age displays both common and dynamic features across human tissues that are influenced by CpG landscape
Source: Genome Biol. 2013 Sep 13;14(9):R102. doi: 10.1186/gb-2013-14-9-r102 (PMC4053985; doi:10.1186/gb-2013-14-9-r102)
Supplement: Additional file 1 — Description of samples used in this study. [file gb-2013-14-9-r102-S1.docx]

Supplemental Table 1. Description of samples used in this study


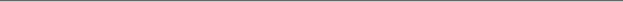


blood^a^ brain^b^ kidney^c^ muscle^d^


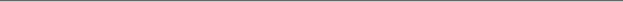


**sample size** 71 78 83 51

**Age^e^ (years)** 55±14.5 22.9±16.6 61.3±14.1 50±17.1

**gender:**

**male** 22 57 51 --

**female** 49 21 32 --

**ethnicity:**

**Caucasian** 58 50 0 0

**African American** 12 2 0 0

**Asian** 1 1 0 0

**unknown** 0 25 83 51

**diagnosis:**

**iron deficient anemia** 59 -- -- --

**anemia, undefined** 7 -- -- --

**normocytic anemia** 5 -- -- --

**brain control** -- 40 -- --

**brain autism** -- 38 -- --


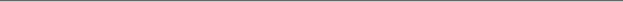


^a^ buffy coat, various anemias

^b^ Autism case and controls collected from Brodmann area 19

^c^ majority collected from cortex outside of matching tumor tissue boundary

^d^ vastus lateralis

^e^ Mean±SD
