# Supplementary material for: Differential DNA methylation with age displays both common and dynamic features across human tissues that are influenced by CpG landscape
Source: Genome Biol. 2013 Sep 13;14(9):R102. doi: 10.1186/gb-2013-14-9-r102 (PMC4053985; doi:10.1186/gb-2013-14-9-r102)
Supplement: Additional file 7 — Supplemental Figures S1 to S24. [file gb-2013-14-9-r102-S7.pdf]

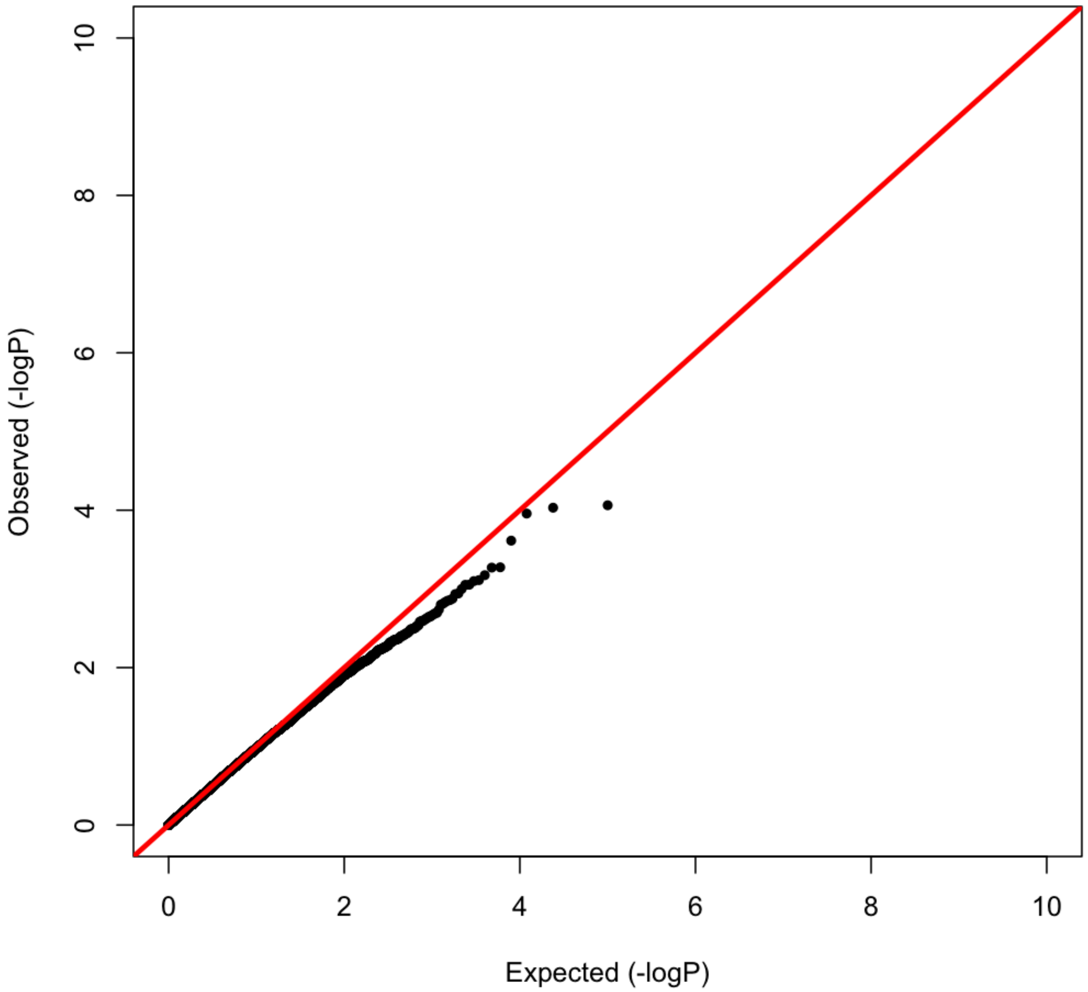

Figure S1. Uniform quantile-quantile (Q-Q) plots of negative log *p*-values associated with our linear regression results for tests between percent methylation and autism.

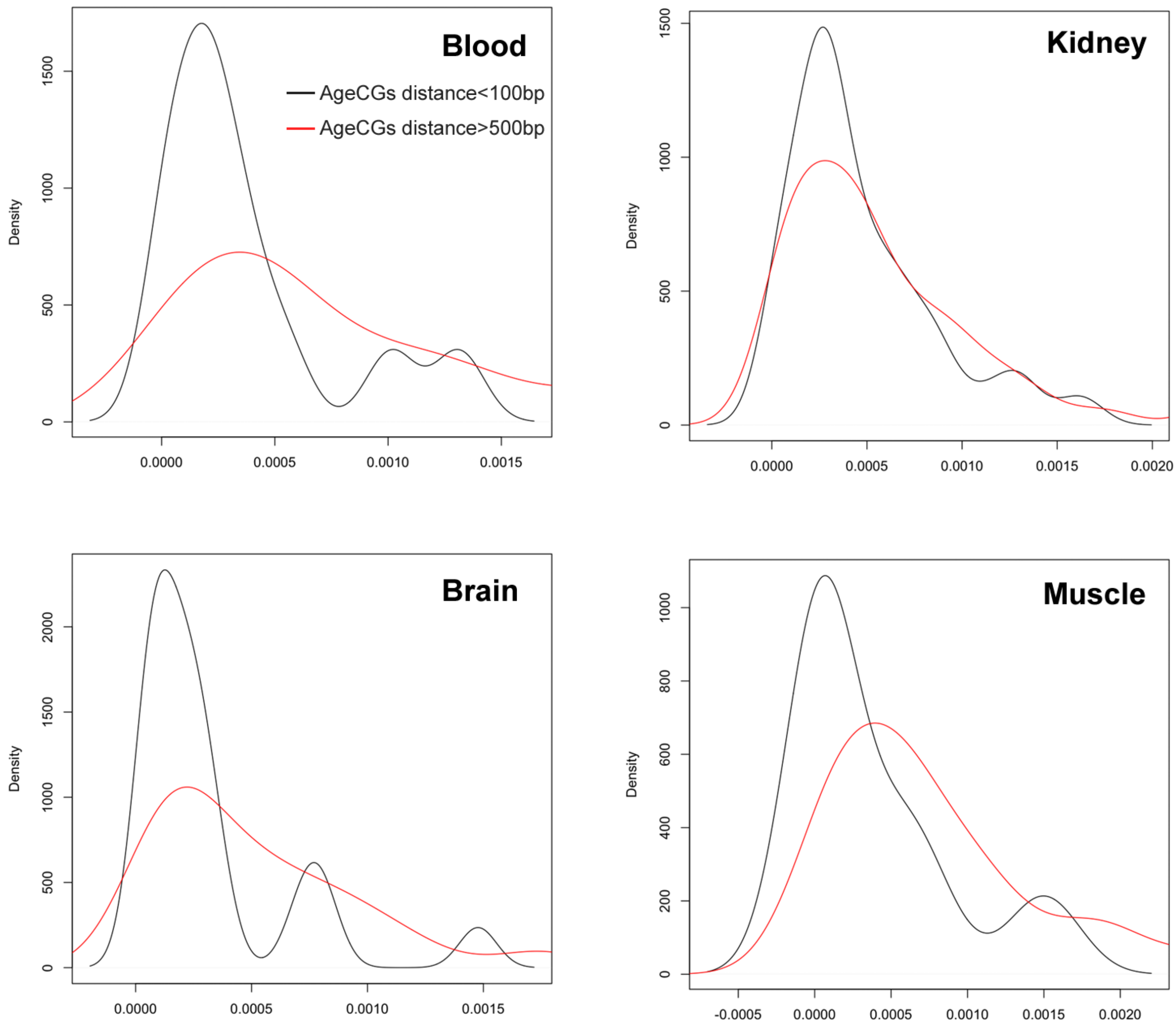

Figure S2. Density curves for absolute value of slope delta between proximal ageCGs that were either less than 100bp in distance from one another or greater than 500 bp.

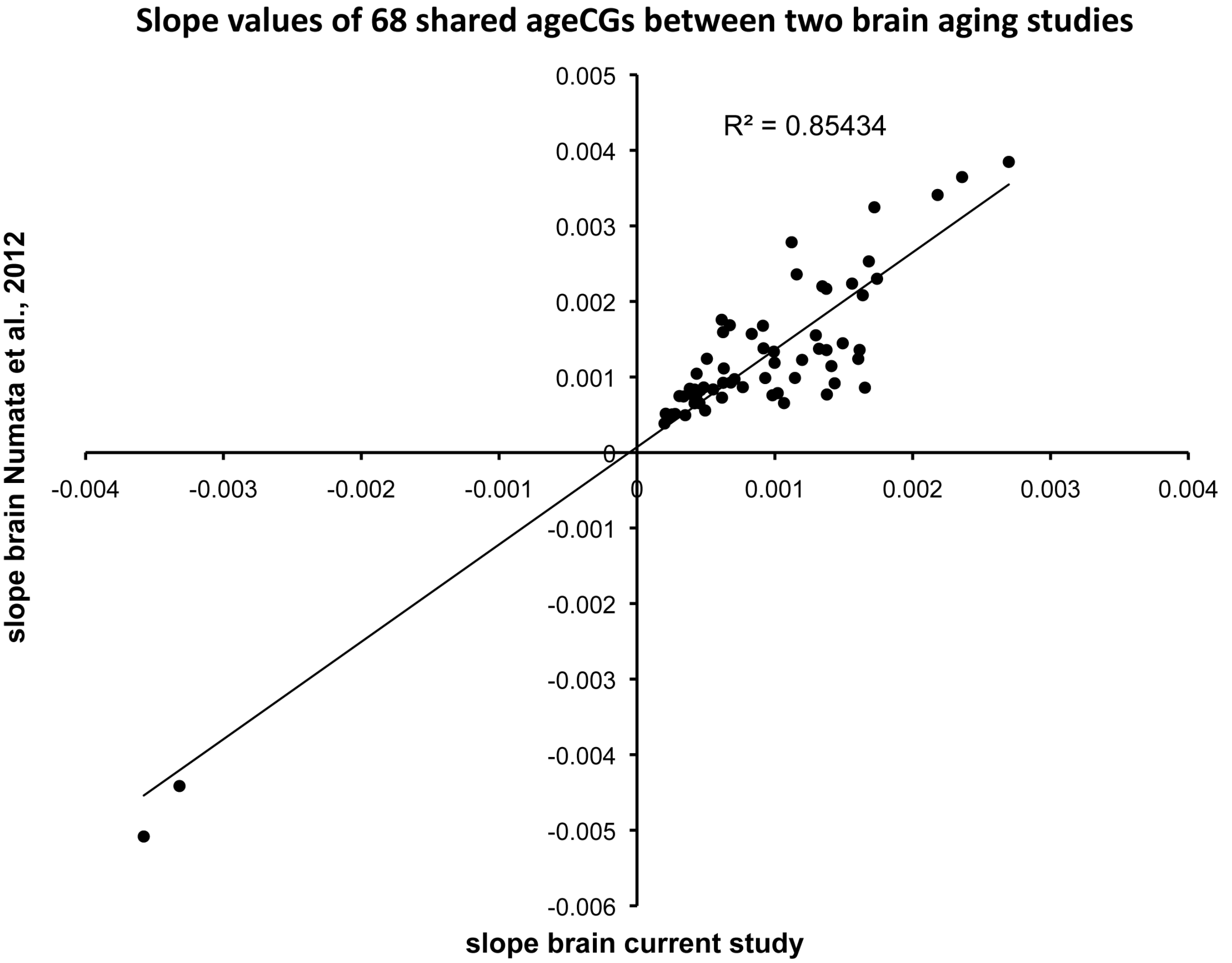

Figure S3. Correlation between slopes of our study and an independent study for CpGs that exhibited strong age-dependent methylation.

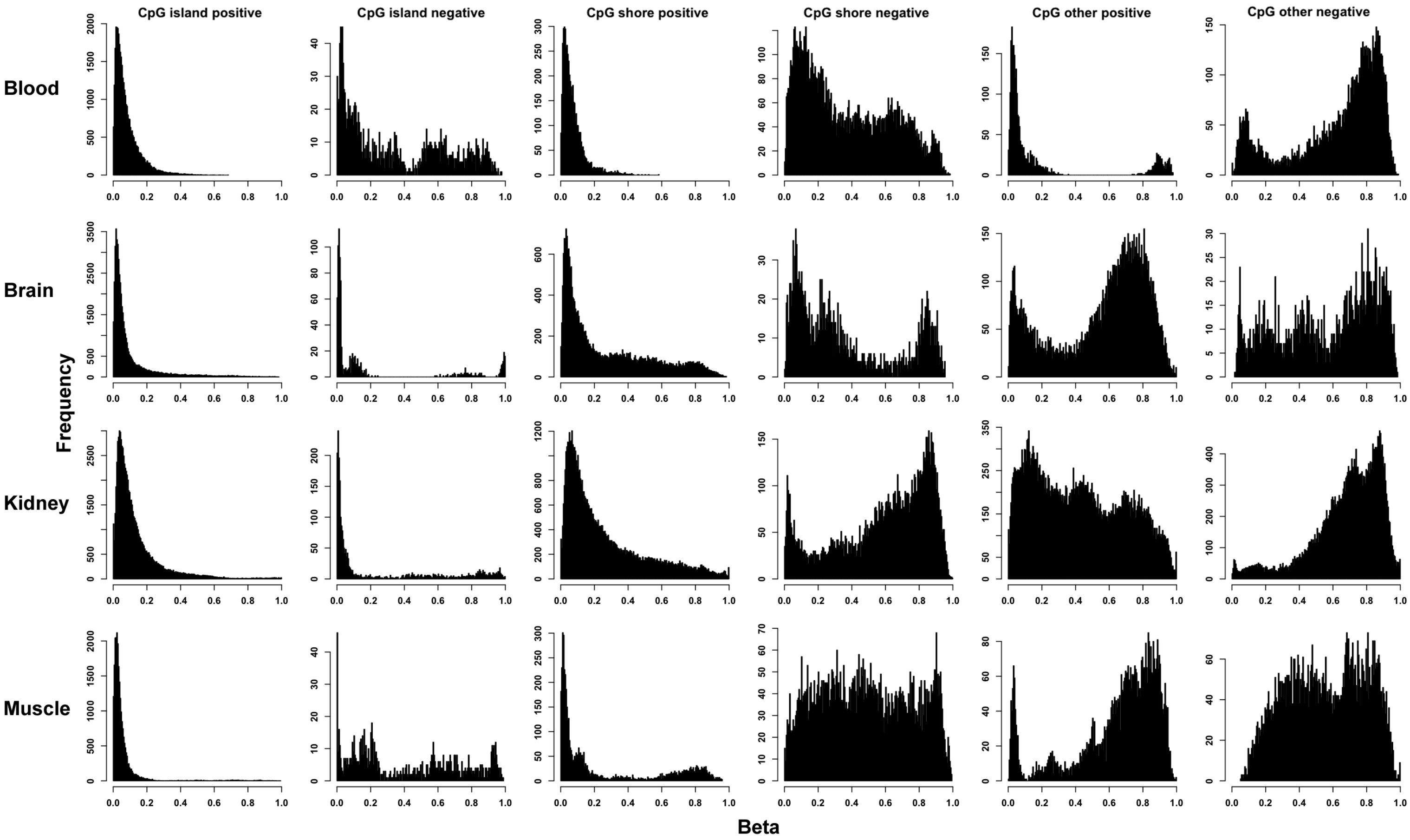

Figure S4. Distributions of β-scores at corresponding positive or negative ageCGs across all samples for each of the 4 tissues grouped according to CGI, CGS, or CGO annotations.

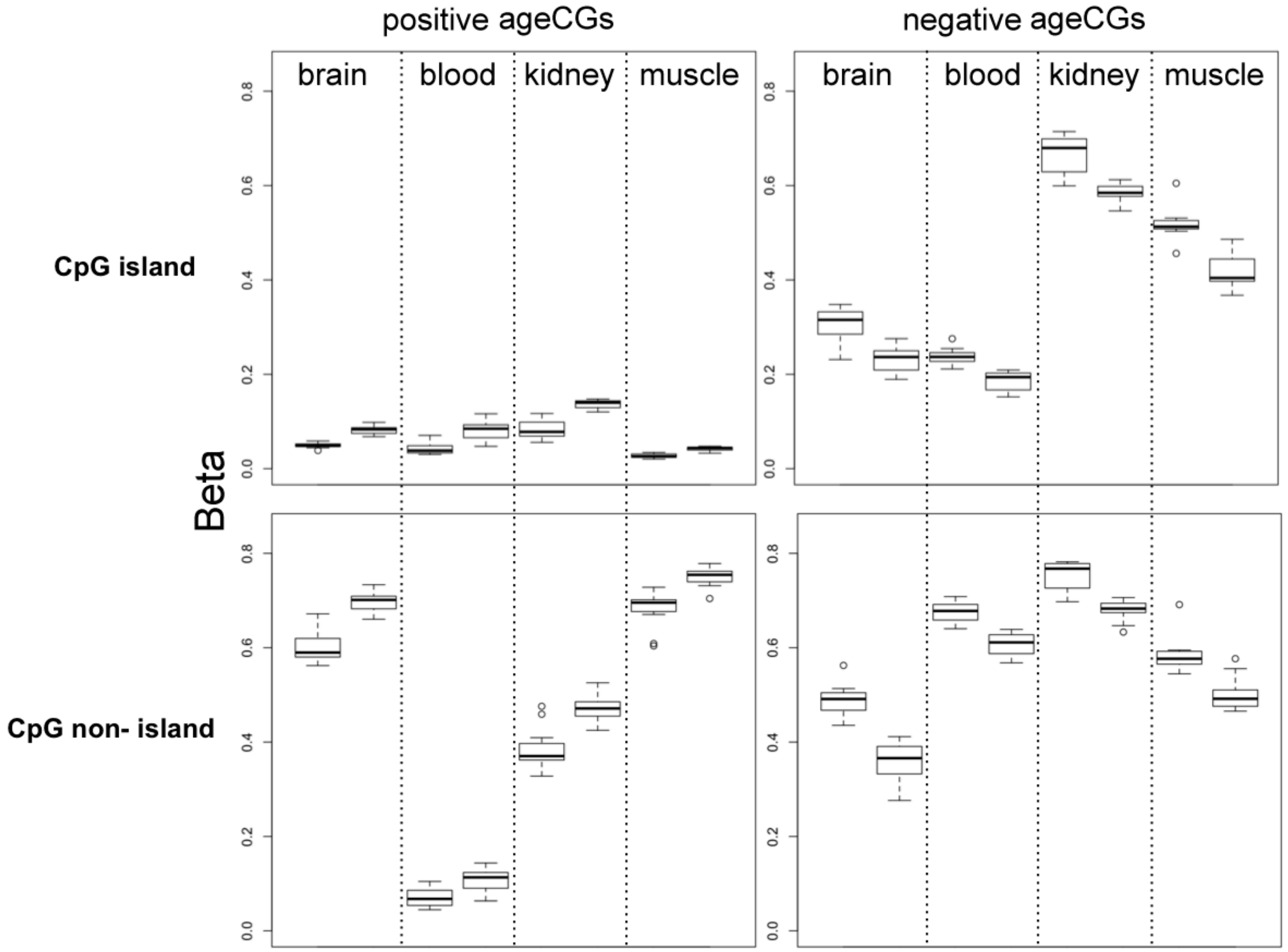

Figure S5. Boxplots depicting β-scores at ageCGs for the 10 youngest and 10 oldest samples from each tissue grouped by positive or negative ageCGs and their position within CGIs or non-CGIs. Dotted lines separate each tissue. Left boxplots for each tissue are β-scores from young samples and right boxplots are from old samples. The least variation in β-scores across tissues was found for positive ageCGs within CGIs.

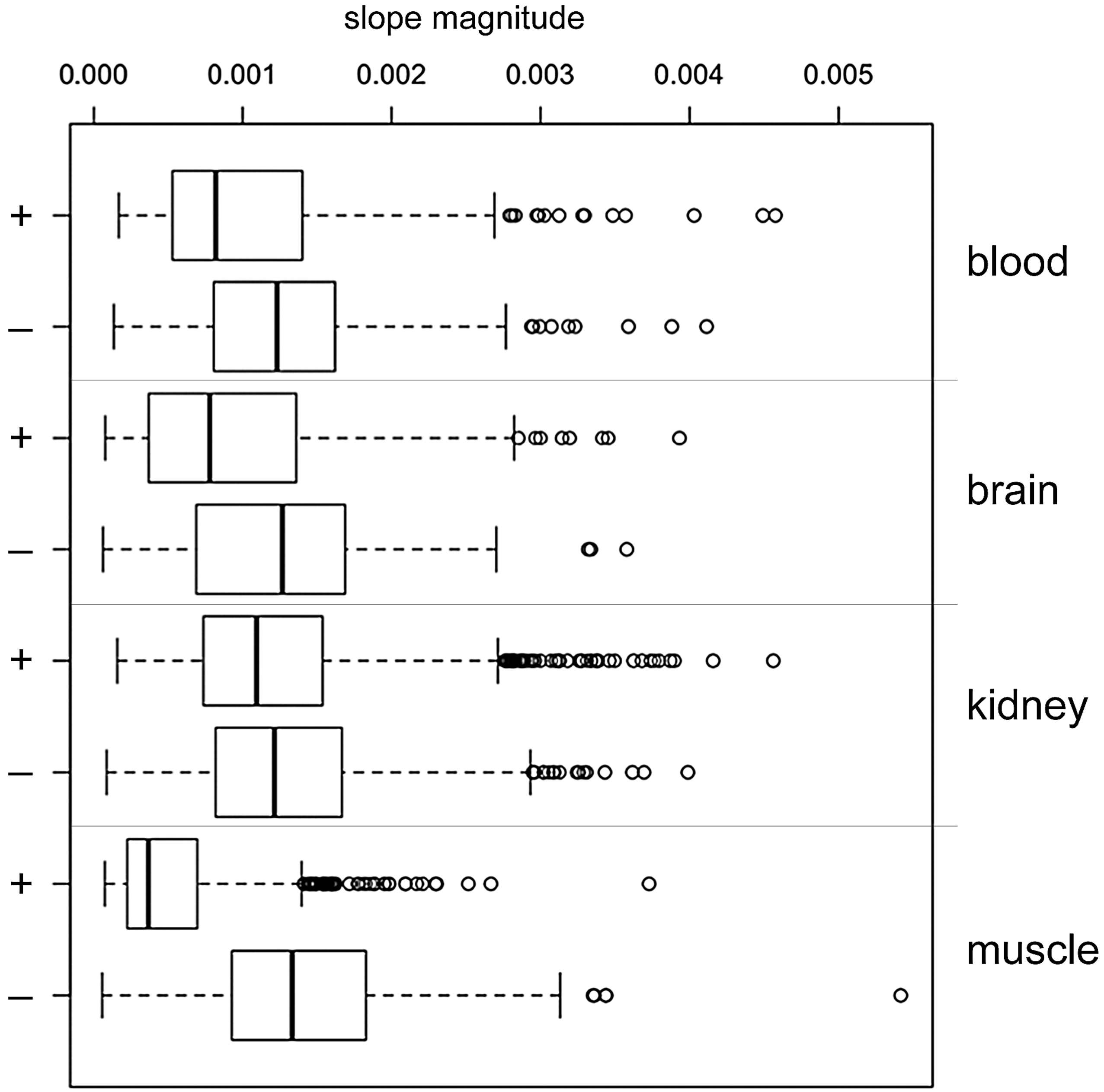

Figure S6. Boxplots depicting absolute value of the slopes (slope magnitude) grouped according to positive (+) or negative (-) ageCGs and by tissue.

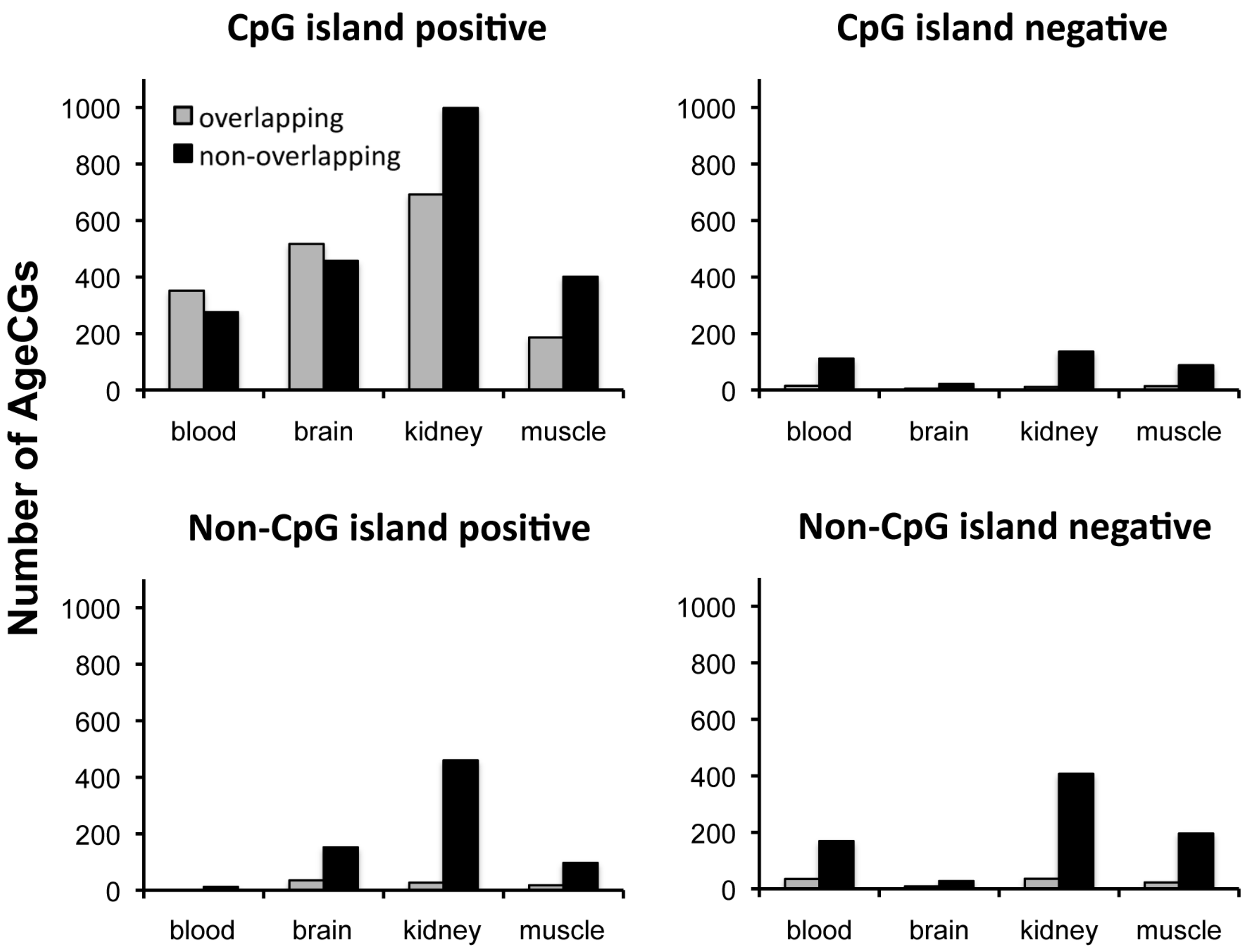

Figure S7. The number of overlapping positive and negative ageCGs between at least 2 tissues subclassified by CpG context. The majority of overlapping ageCGs between at least 2 tissues are positioned within CGIs and showed a positive association with age.

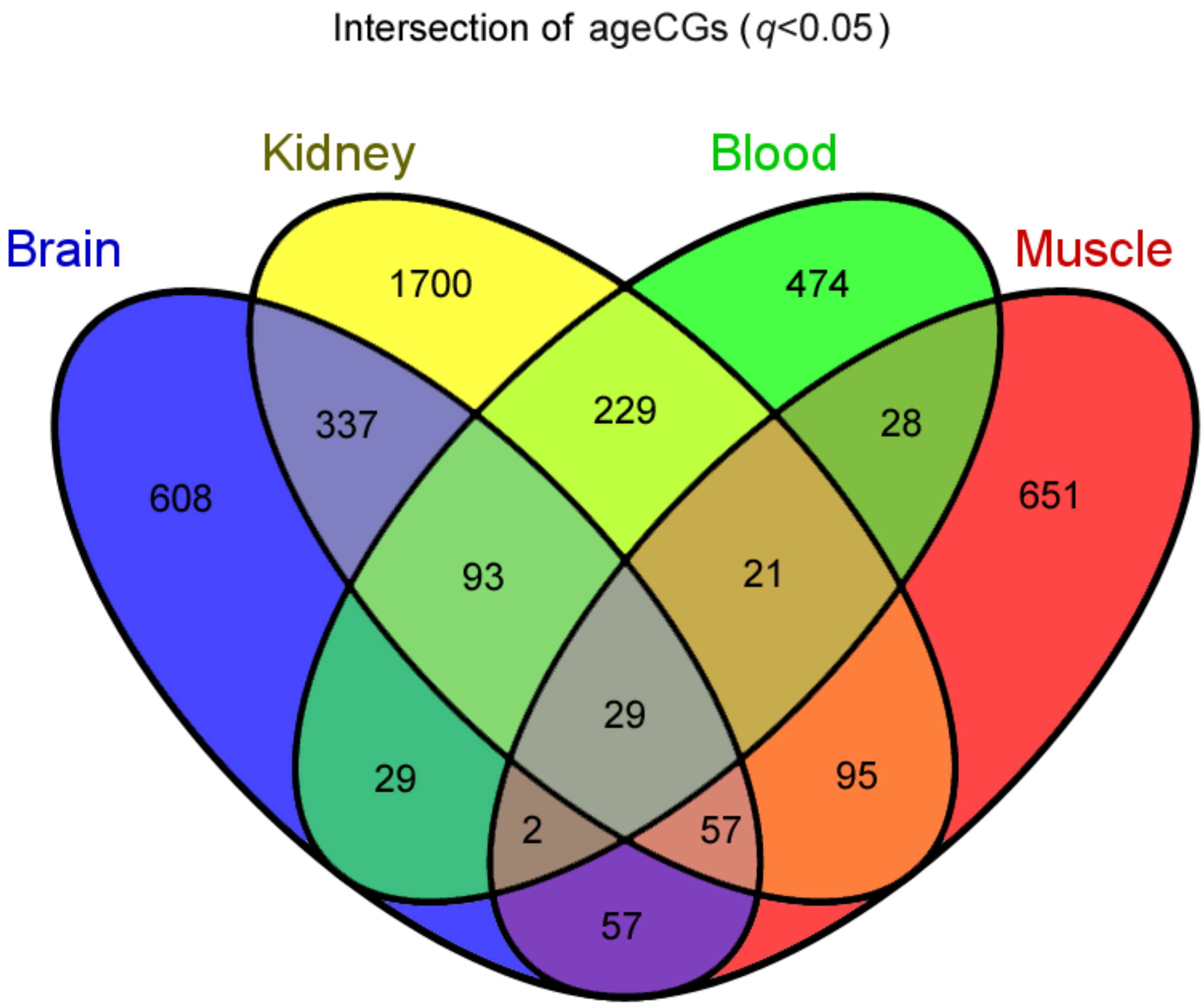

Figure S8. 4-way Venn diagram depicting the intersection of ageCGs across all tissues.

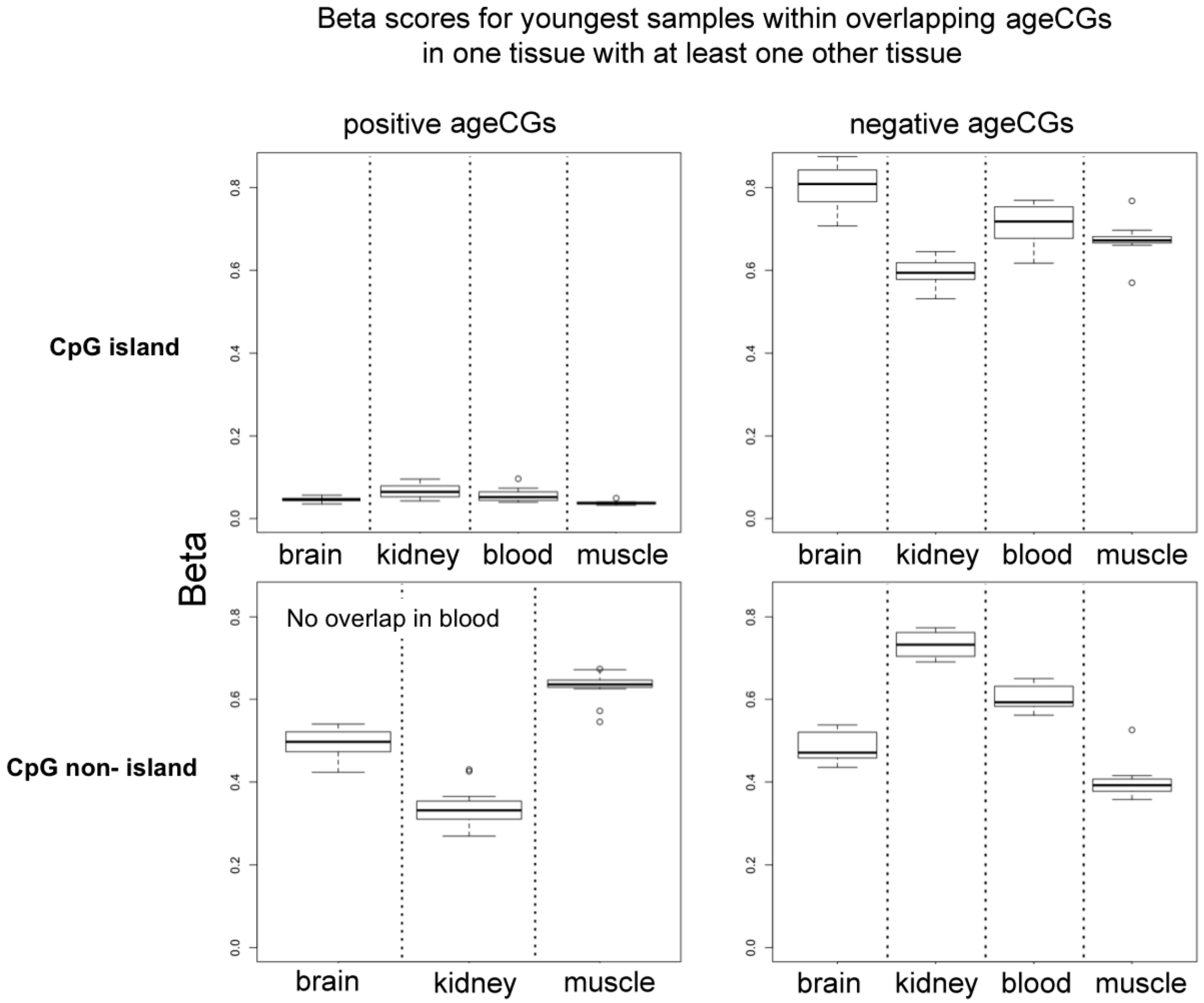

Figure S9. Boxplots depicting beta scores at overlapping ageCGs for the 10 youngest samples in each tissue grouped according to positive or negative ageCGs within CGIs or non-CGIs. Greater variation is observed in β-scores across young samples and tissues for overlapping negative ageCGs positioned within non-CGIs compared to positive ageCGs within CGIs.

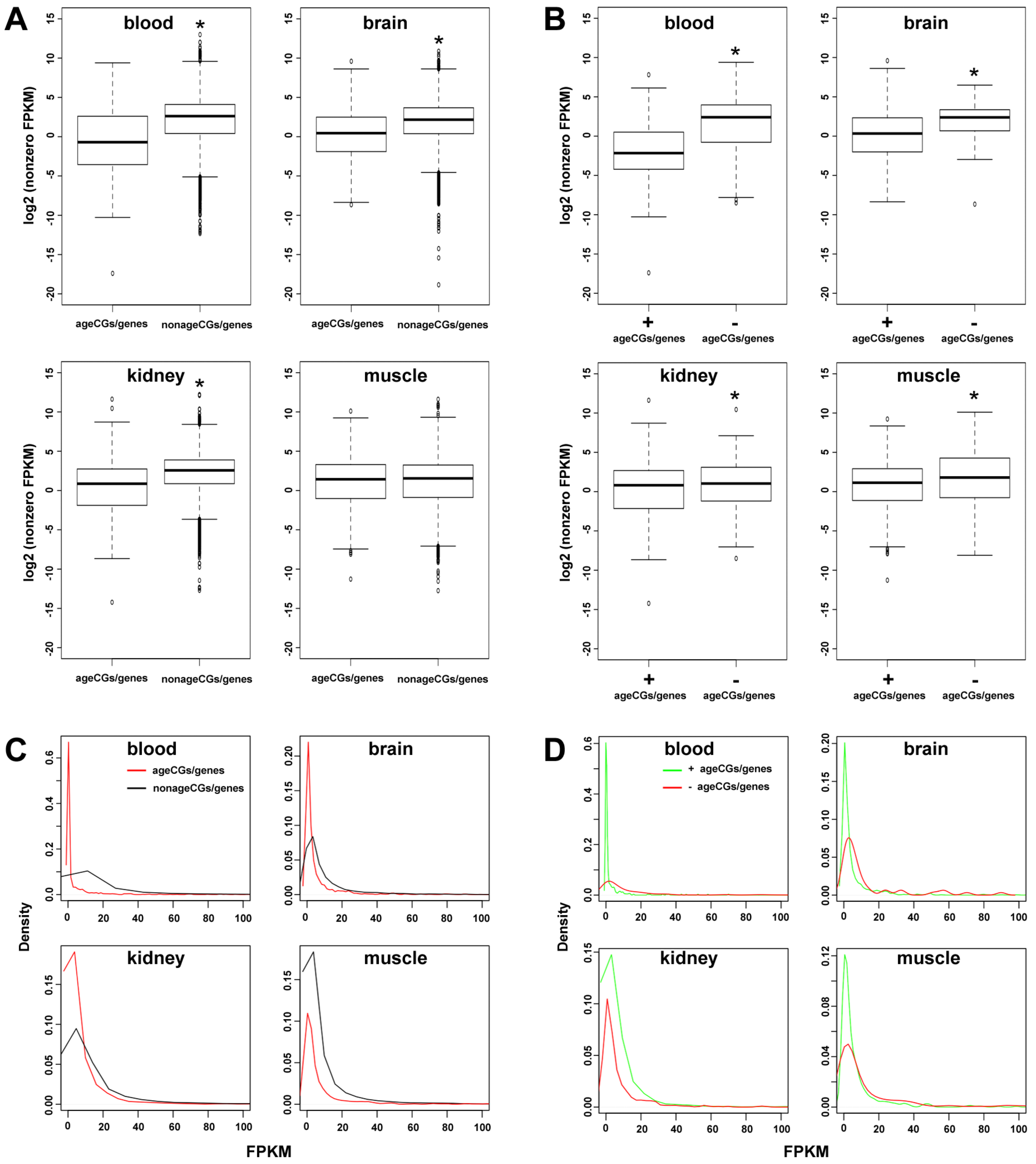

Figure S10. Boxplots depicting corresponding FPKM values for total ageCGs/genes, nonageCGs/genes, positive and negative ageCGs/genes for all tissues. **(a)** Comparisons of ageCGs/genes and nonageCGs/genes for each tissue showed significantly higher gene expression levels (nonzero FPKM) for nonageCGs across all tissues, except for skeletal muscle (asterisks; Mann-Whitney U, $p<2.2\times10^{-16}$). **(b)** Comparisons of positive and negative ageCGs/genes showed significantly higher gene expression levels for negative ageCGs/genes (asterisks; Mann-Whitney U, blood, $p<2.2\times10^{-16}$; brain, $p=1.3\times10^{-5}$; kidney, $p=0.006$; muscle, $p=0.0026$). **(c)** Distributions of FPKM values less than 100 for ageCGs/genes and nonageCGs show a larger proportion of values closer to zero for ageCGs/genes. **(d)** Distributions of FPKM values less than 100 for positive and negative ageCGs/genes show a larger proportion of values closer to zero for positive ageCGs/genes (with the exception of kidney).

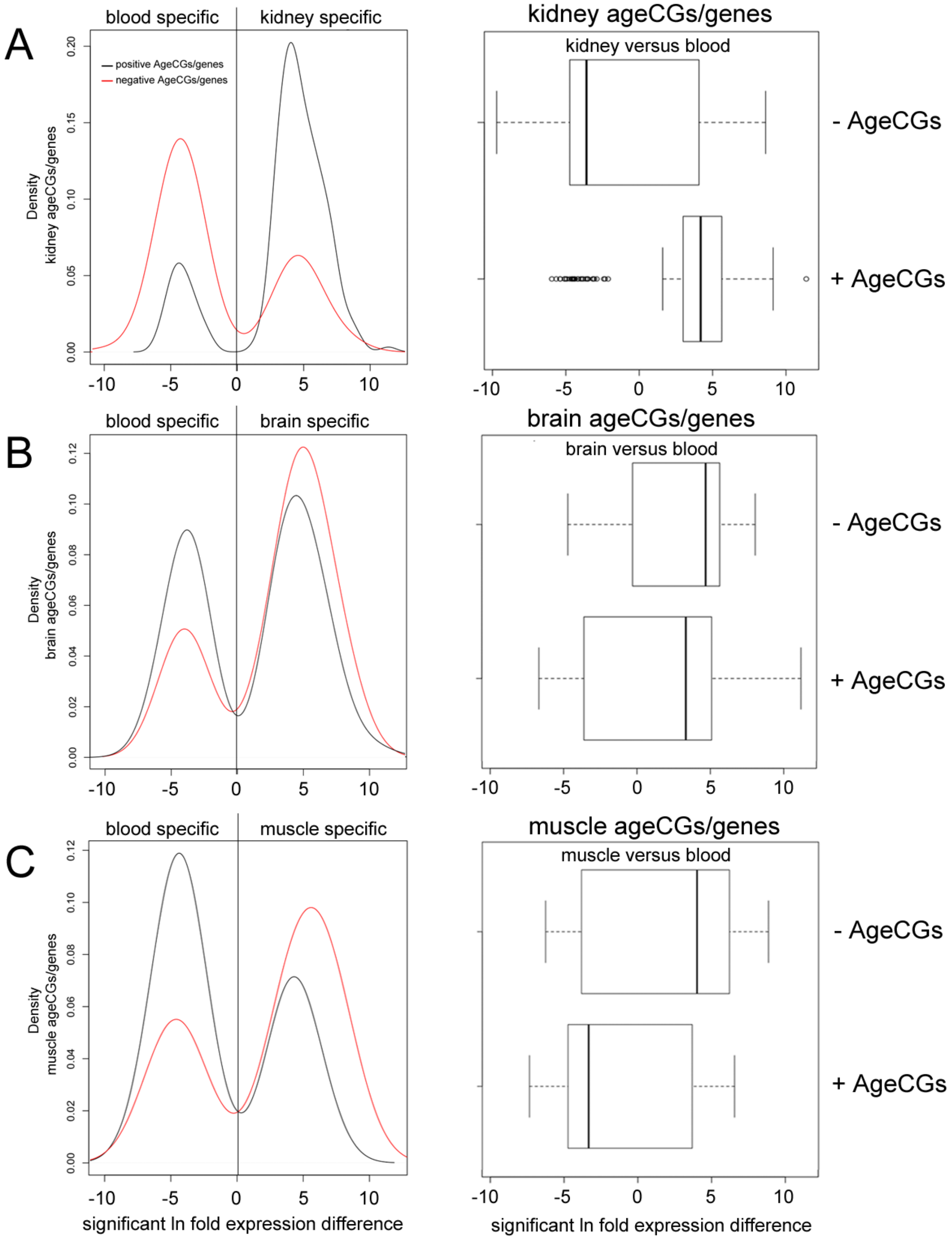

Figure S11. Significant fold gene expression differences between **(a)** kidney and blood **(b)** brain and blood and **(c)** muscle and blood for positive and negative kidney ageCGs/genes, brain ageCGs/genes, and muscle ageCGs/genes, respectively. Significant gene expression differences were determined by Student's t-tests with Cufflinks software. Higher blood-specific expression levels were associated with **(a)** kidney negative ageCGs/genes compared to **(b)** brain and **(c)** muscle negative ageCGs/genes.

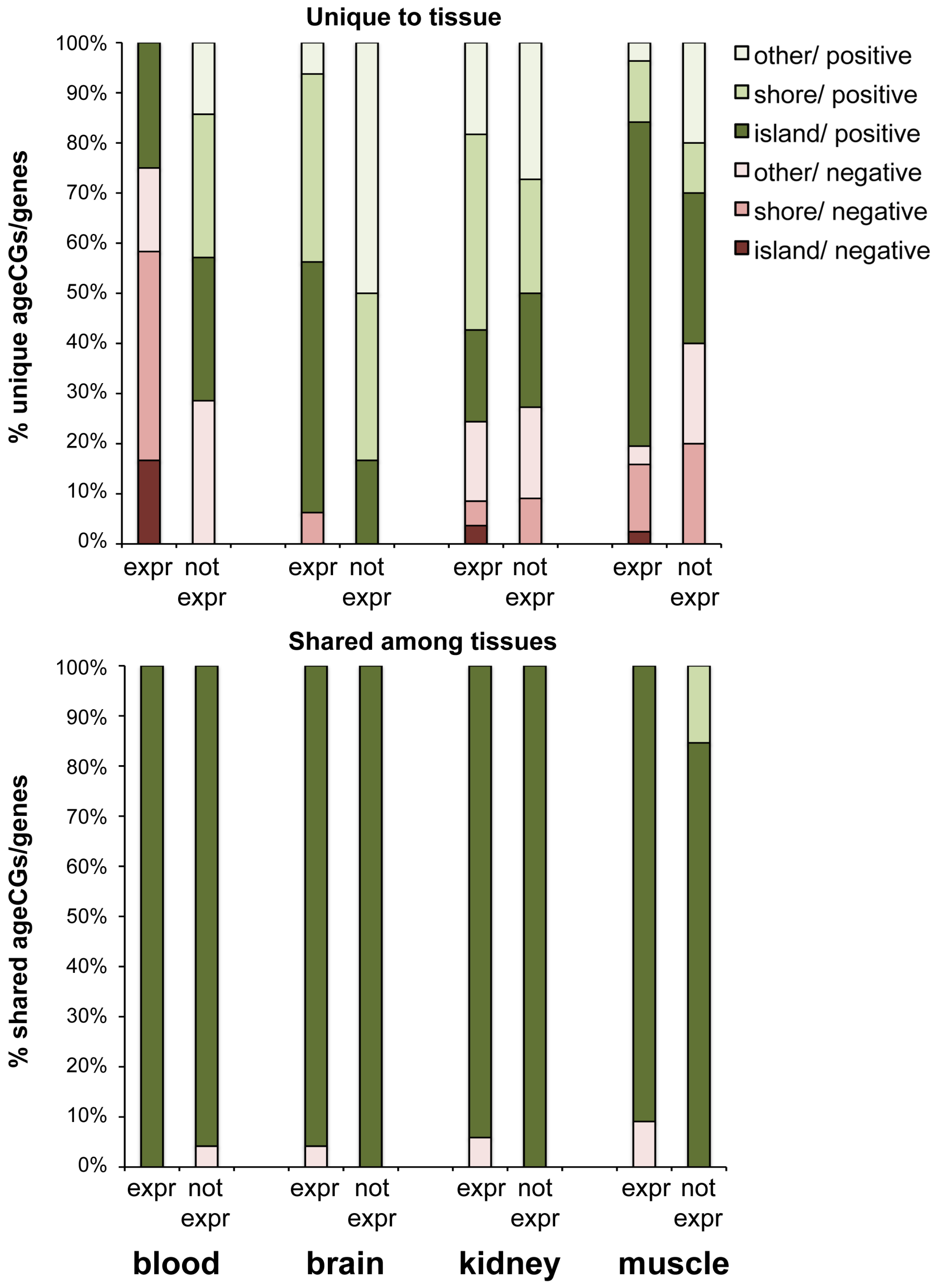

Figure S12. Percentages of unique and shared ageCGs/genes across tissues subclassified according to expression, CpG context, and positive or negative association with age. Regardless of affiliation with expressed genes, shared ageCGs/genes are almost all found within CpG islands bearing positive associations with age. Expr, expressed; not expr, not expressed.

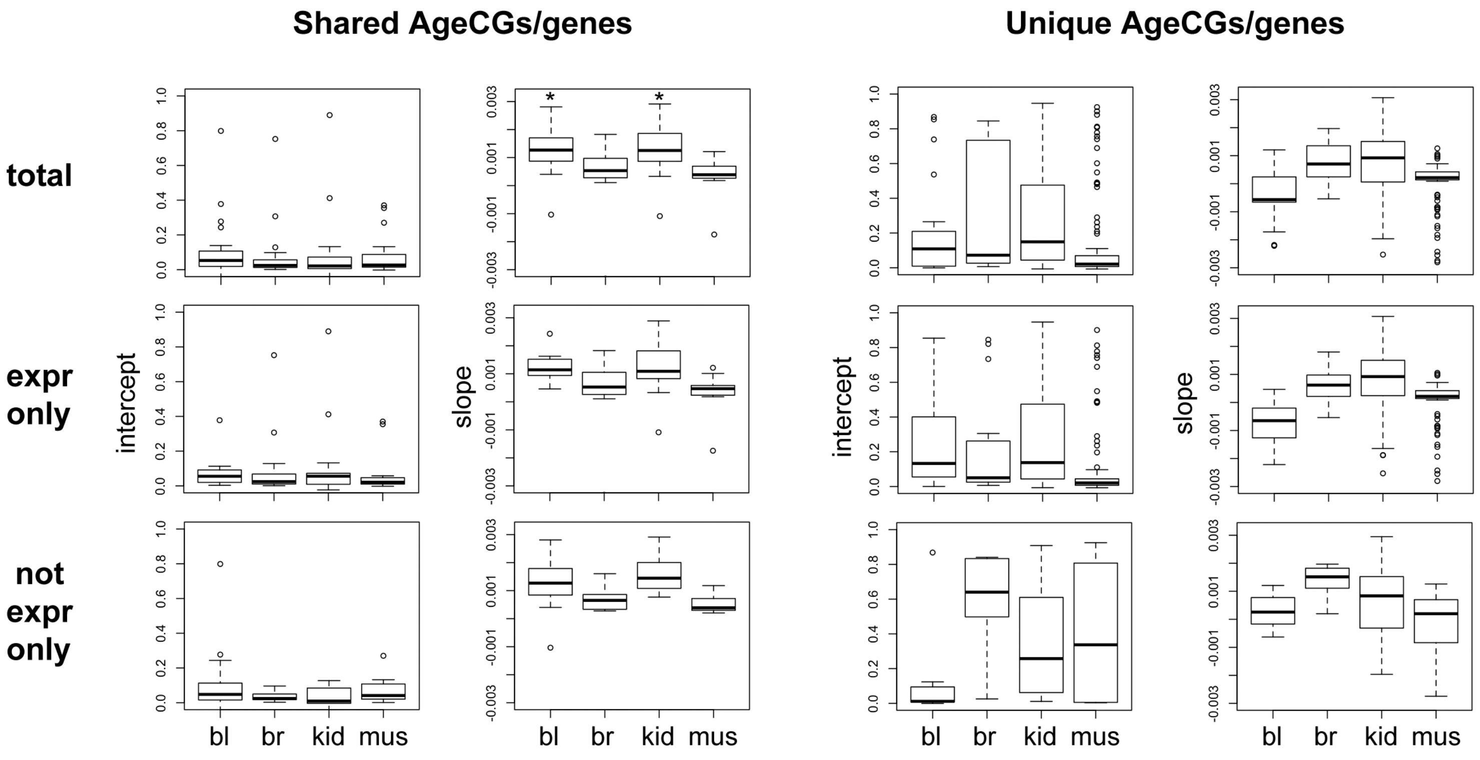

Figure S13. Boxplots depicting intercepts and slopes for shared or unique ageCGs/genes across tissues subclassified by affiliation with expressed or not expressed genes within respective tissues. More variation in intercepts and slopes are observed in unique compared to shared ageCGs/genes. bl, blood; br, brain; kid,kidney; mus, skeletal muscle.

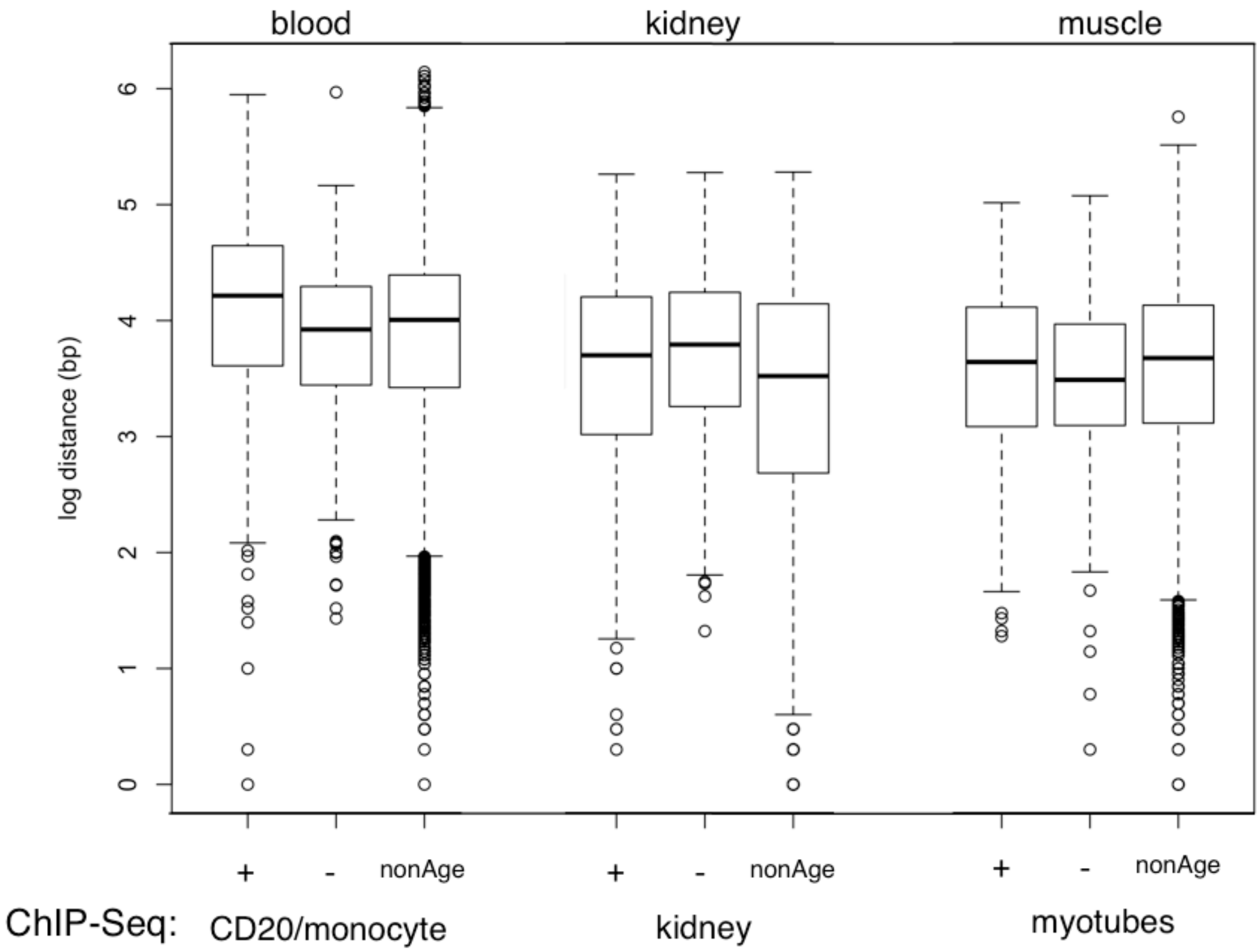

Figure S14. Boxplots depicting $\log_{10}$ distance between positive ageCGs (+), negative ageCGs (-), nonageCGs, and nearest CTCF binding sites for blood (CD14+ and CD20+ cells; combined peaks), kidney tissue, and myotubes.

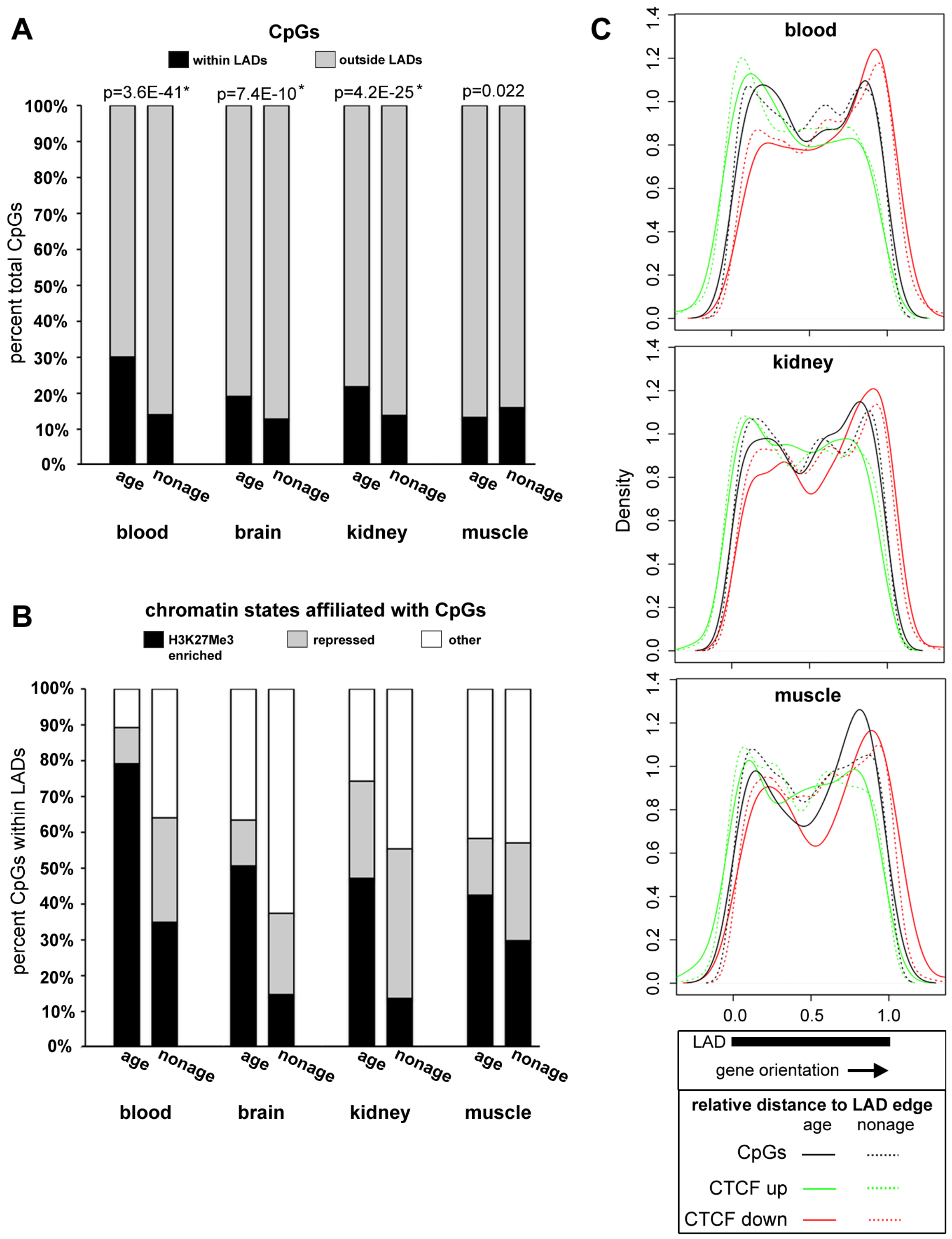

Figure S15. AgeCGs are enriched within LADs. **(a)** Approximately 19-30% of total ageCGs in each tissue are significantly enriched within LADs compared to nonageCGs, with the exception of skeletal muscle. *P*-values resulting from Pearson's Chi-squared tests for count data within or outside of LADs are shown above the bars. Asterisks near *p*-values indicate significant differences between ageCGs and nonageCGs within each tissue that meet Bonferroni correction. **(b)** LADs containing ageCGs are enriched in H3K27Me3-related chromatin states as shown by the percentages of ageCGs within LADs grouped according to chromatin states and compared to nonageCGs. **(c)** CpGs within LADs and tissue-specific CTCF binding sites are positioned on LAD edges as shown by the distribution of genomic distance of ageCGs or CTCF sites within LAD edges subtracted from the LAD start site and divided by total LAD size. Distributions were adjusted to gene orientation and mirrored to show genes oriented toward the middle of a LAD or away from a LAD both enriched on boundaries. Legend depicts relative distance within a LAD, normalized by LAD size from 0 (start) to 1 (end). Bifurcated distributions indicate CpGs and CTCF binding sites positioned near LAD edges. Both upstream (green lines) and downstream (red lines) CTCF binding sites in relationship to the CpGs (black lines) are depicted. Dotted lines indicate nonageCGs.

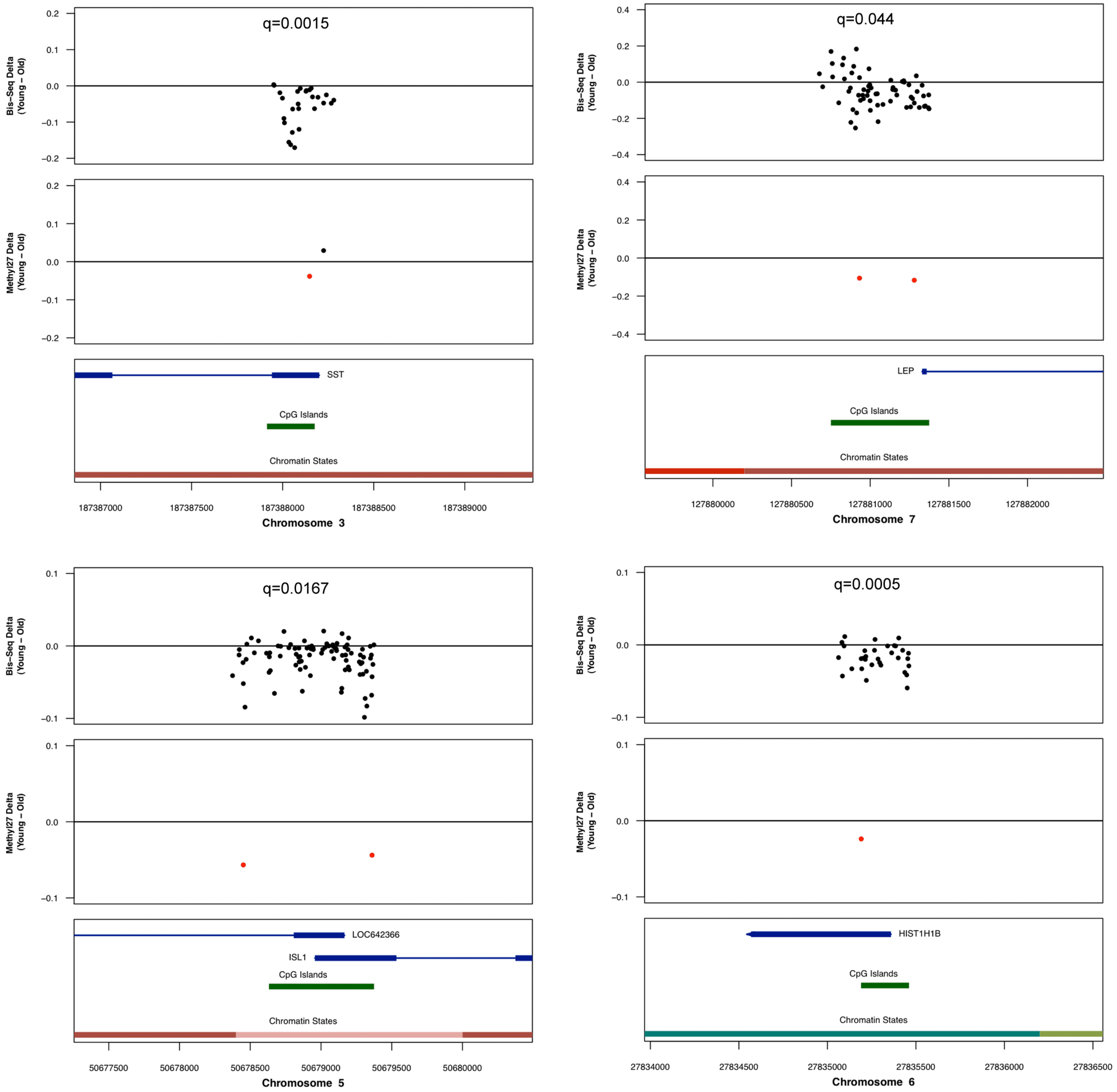

Figure S16. Additional genome plots of significant widespread age effects according to mixed linear model results for Bis-Seq data similar to those shown in Figure 5c. Points depict delta in median percent methylation values between the 9 youngest and 10 oldest samples at CpGs covered by Bis-Seq (topmost plots of each panel) compared to delta β−score values from the same set of 9 youngest and 10 oldest samples at CpGs covered by Methylation27 arrays (middle plots in each panel). FDR (q) values depicted above plots shown indicate significance level of a widespread age effect within these targets that covered multiple CpGs. Red points represent significant CpGs associated with age identified by the all kidney sample linear regression analysis on Methylation27 array data, but delta value shown is only for the same 19 samples that were sequenced.Bottommost plots in each panel depict labeled gene regions (blue boxes - exons; blue lines - introns), CpG islands (green boxes), and fetal kidney chromatin states (colors defined by inset legend on right panels correspond with definitions in Figure 4).

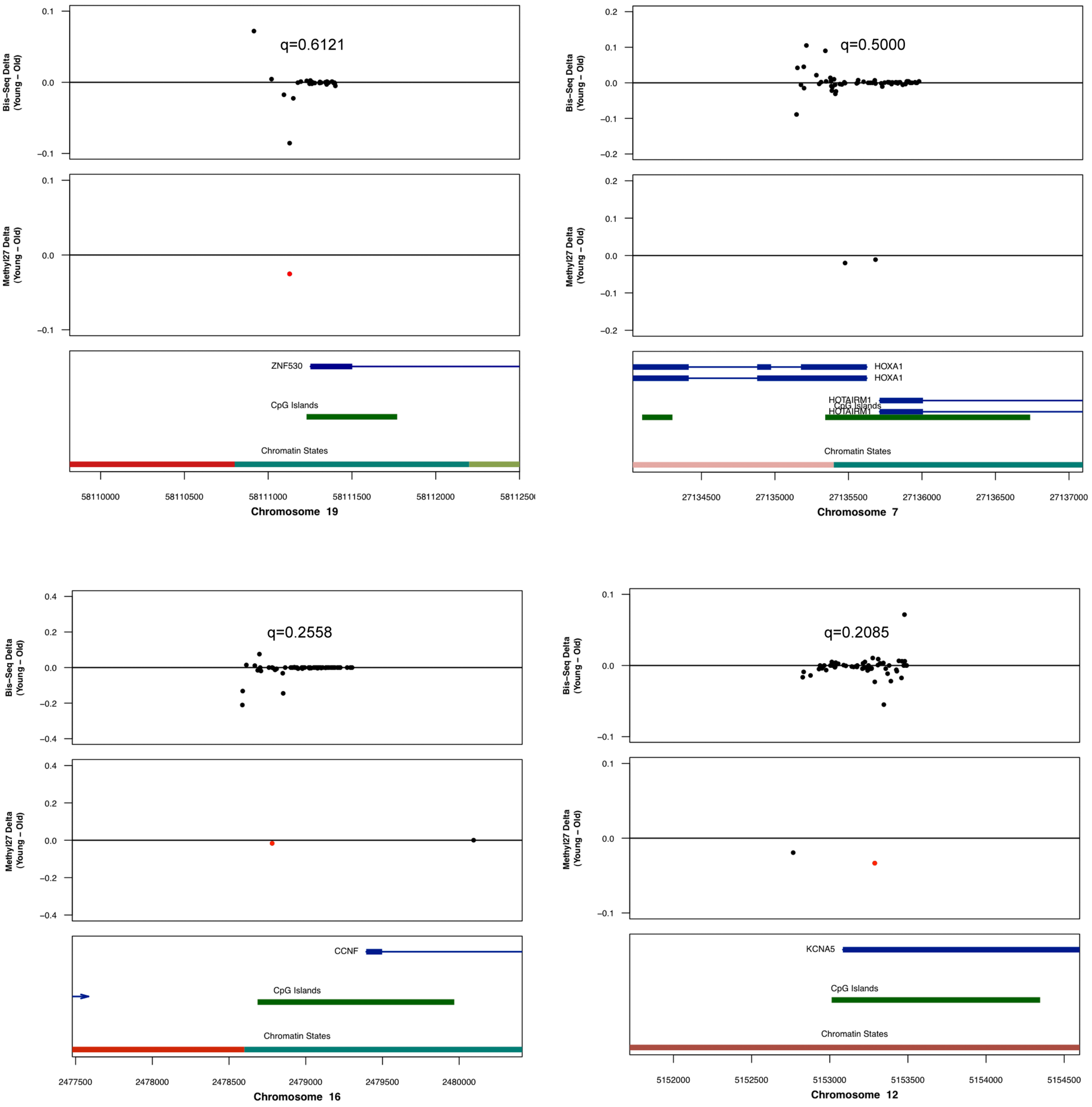

Figure S17. Additional genome plots of targets that did not exhibit significant widespread age effects according to mixed linear model results for Bis-Seq data across multiple CpGs similar to those shown in Figure 5d. The age effect appears to be isolated to a few CpGs within each target. Red points represent significant CpGs associated with age identified by the all kidney sample linear regression analysis on Methylation27 array data, but delta value shown is only for the same 19 samples that were sequenced.See legend of previous figure.

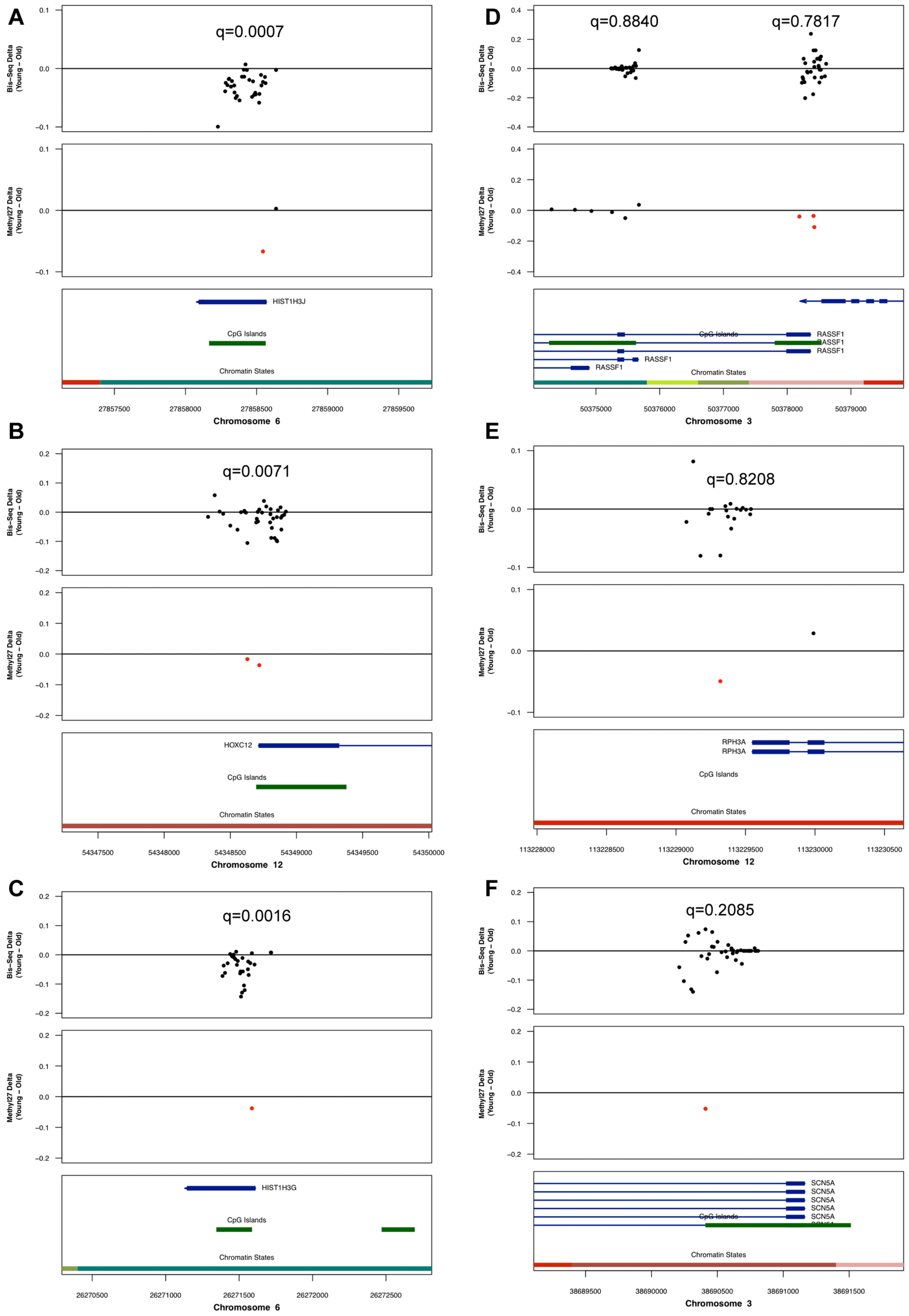

Figure S18. Additional genome plots of targets (a-c) that exhibited significant widespread age effects according to mixed linear model results for Bis-Seq data across multiple CpGs, but were not significant with age within methyation27 CpGs where regression was only across the 19 samples alone. Red points represent significant CpGs associated with age identified by the all kidney sample linear regression analysis on Methylation27 array data, but delta values shown are only for the same 19 samples that were sequenced. (d-f) Bis-Seq targets that exhibited abrupt oscillation in the direction of the age effect in neighboring CpGs.

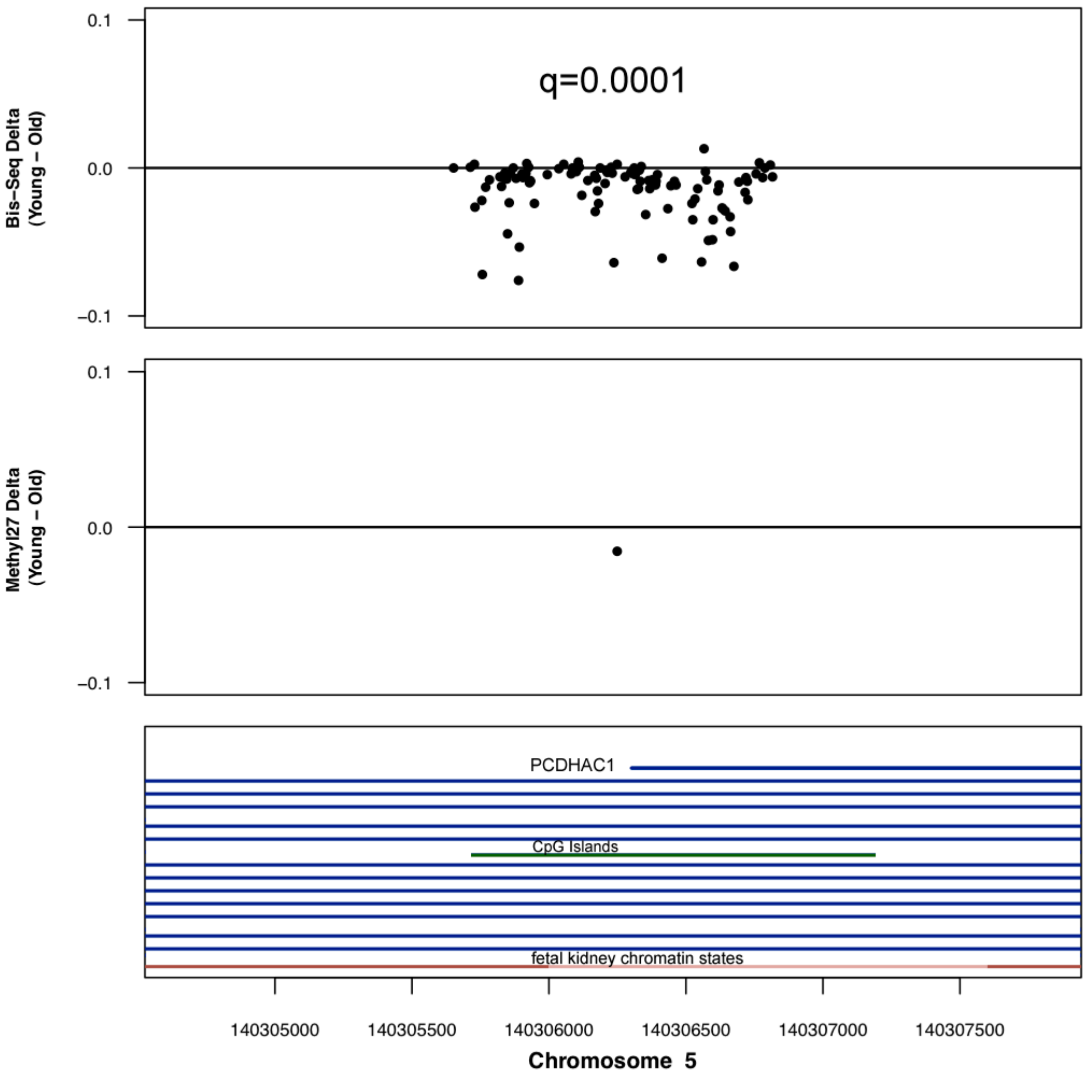

Figure S19. Genome plot depiction of a negative control target (did not contain any ageCGs by methylation 27) that exhibited significant widespread age effects according to mixed linear model results for Bis-Seq data across multiple CpGs.

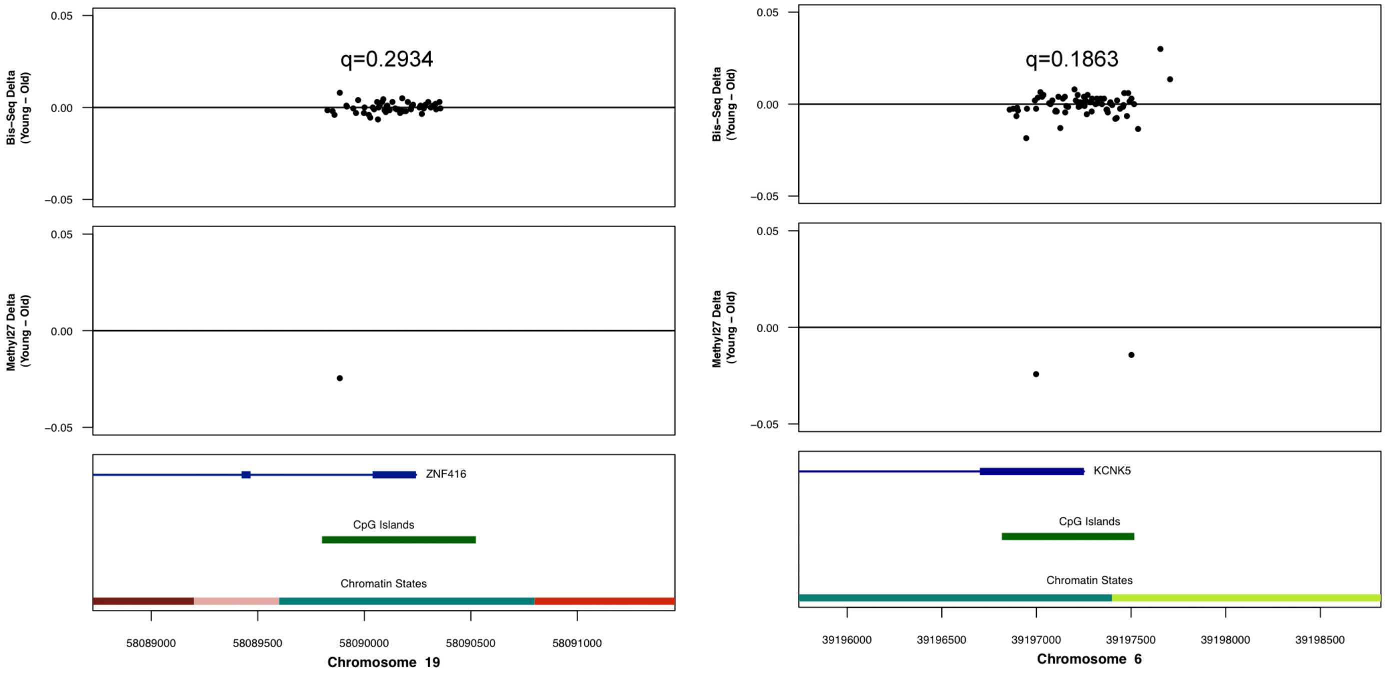

Figure S20. Genome plot depiction of negative control targets (did not contain any ageCGs by methylation 27) that also did not exhibit significant widespread age effects according to mixed linear model results for Bis-Seq data across multiple CpGs.

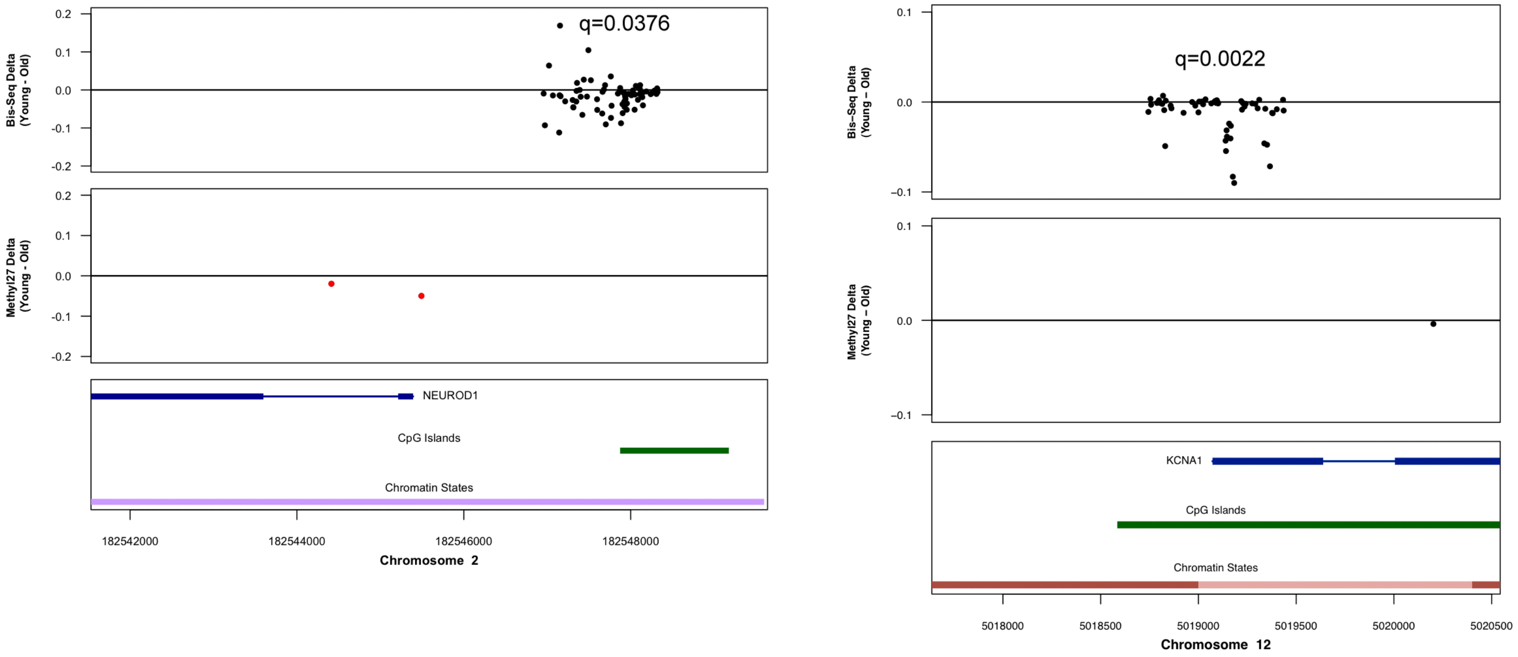

Figure S21. Genome plot depiction of Bis-Seq targets not centered over methylation 27 CpGs that exhibited significant widespread age effects according to mixed linear model results for Bis-Seq data across multiple CpGs.

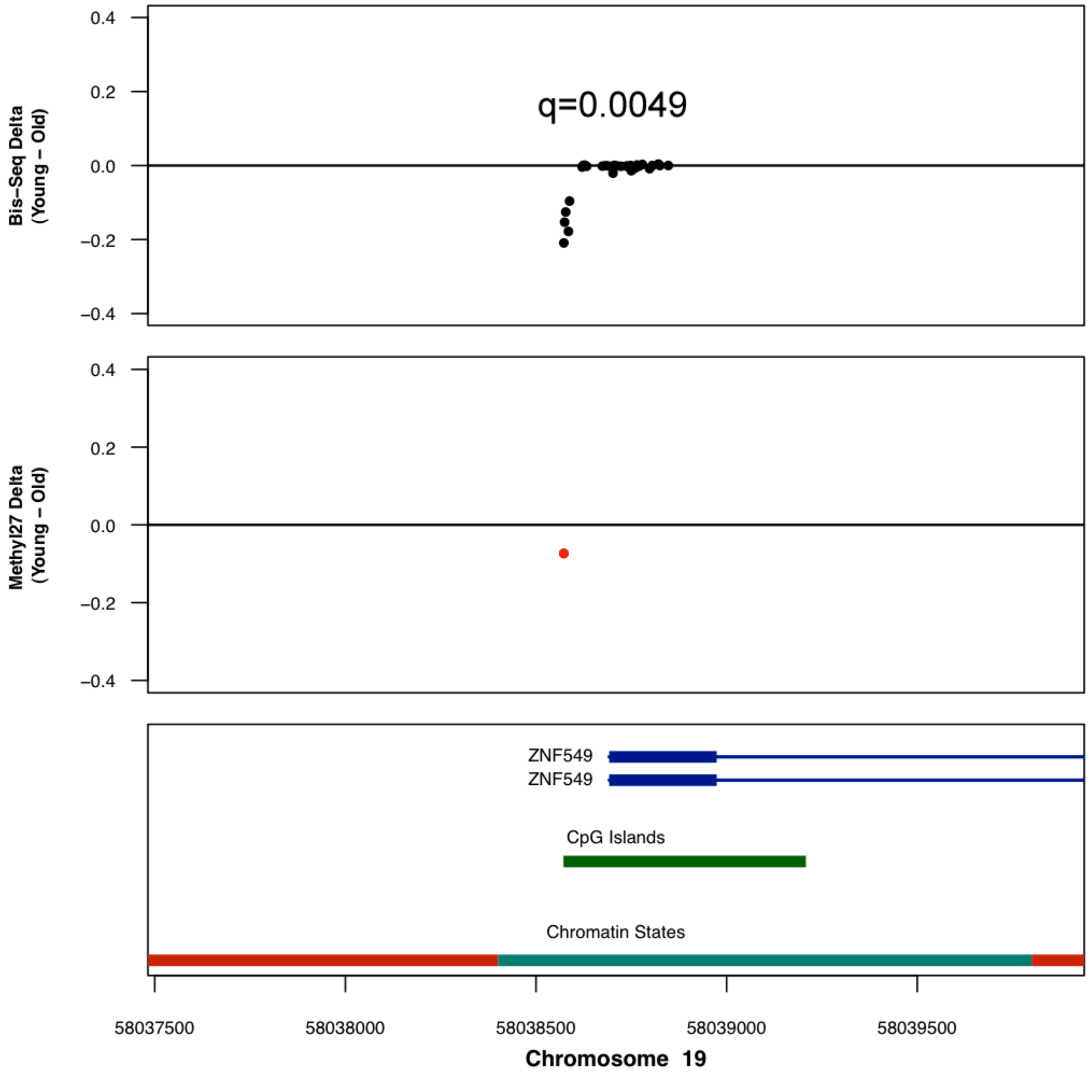

Figure S22. Genome plot depiction of a Bis-Seq target that captured an age effect “border.”

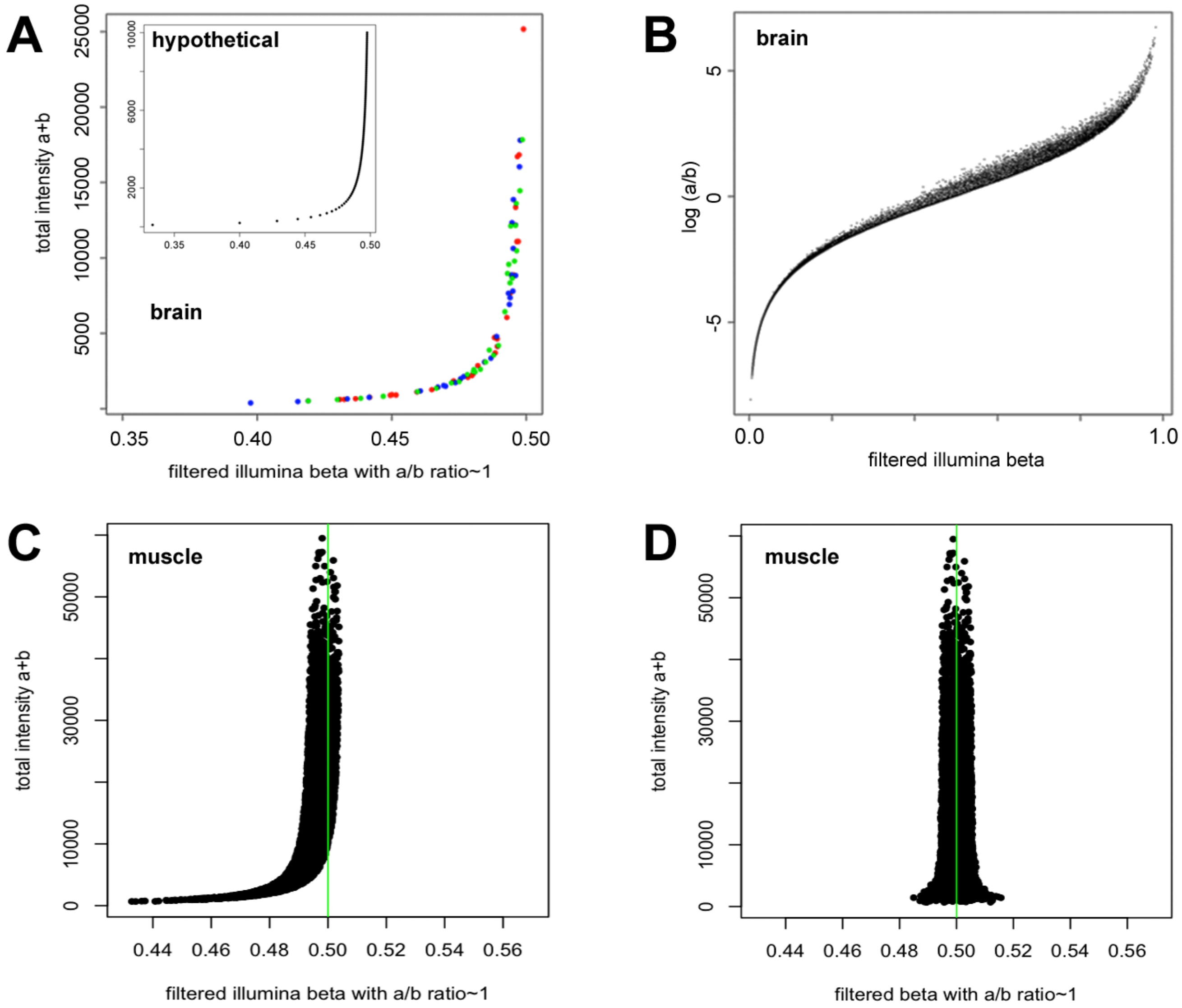

Figure S23. Illumina β-scores calculated with 100 in the denominator for data containing A/B intensity ratios near 1 shift β-scores away from their expected value (0.5). **(a)** Hypothetical Illumina β-scores plotted against total A+B probe intensities for hypothetical A/B ratios near 1 containing increasing equal A and B probe intensity values in increments of 100; Actual Illumina β-scores from brain tissue plotted against total intensities for β-scores calculated from data containing A/B ratios near 1 (blue, red, and green color represent 3 different samples). **(b)** Illumina β-scores from all brain samples plotted against log A/B ratios for each respective β-score shows a distortion of β-score values near ratios close to 1. **(c)** Actual Illumina β-scores from all muscle samples plotted against total intensities for β-scores calculated from data containing A/B ratios near 1 (0.98 to 1.02). **(d)** The same generated plot as in (c) except with omission of 100 in the denominator for β-score calculation.

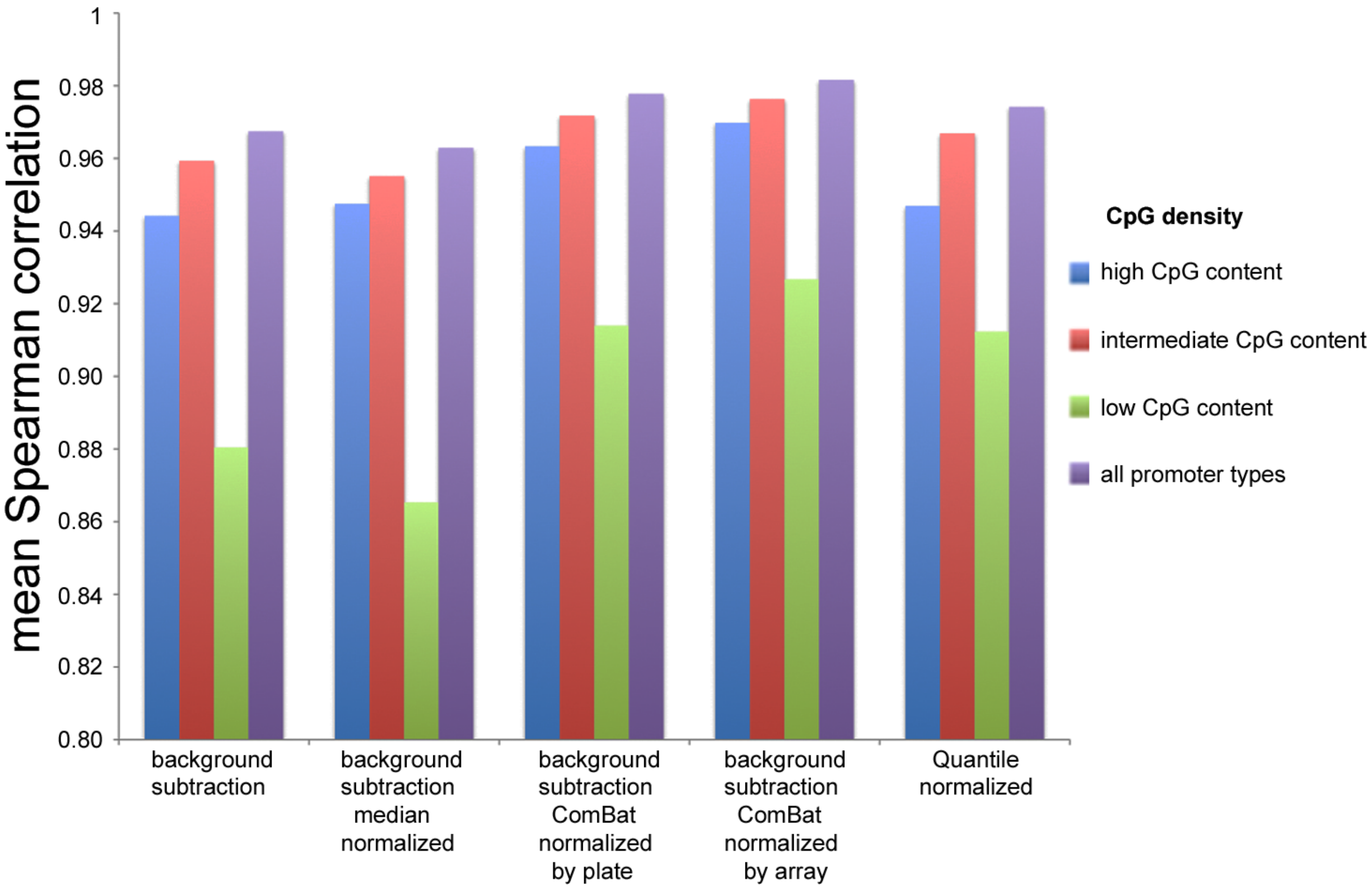

Figure S24. Empirical Bayes nonparametric based normalization by ComBat using sentrix barcode (array ID) as batches generated the highest average Spearman rank correlation coefficients among 23 replicate brain samples compared to other batch normalization methods for β-scores. Classification of β-scores generated from promoter regions with differing numbers of CpGs according to Weber et al., 2007 shows that ComBat batch normalization produced the strongest correlation values across all types of promoters.
